# First organocatalytic, diastereoselective synthesis of tRNA wobble nucleosides: (*R*)- and (*S*)-methoxycarbonylhydroxymethyluridines (mchm^5^Us) and their acid analogues (chm^5^Us)†

**DOI:** 10.1039/d5ra04760a

**Published:** 2025-07-29

**Authors:** Tomasz Bartosik, Agnieszka Dziergowska, Blazej Kowalski, Grazyna Leszczynska

**Affiliations:** a Lodz University of Technology, Institute of Organic Chemistry Zeromskiego 116 90-924 Lodz Poland tomasz.bartosik@p.lodz.pl

## Abstract

A diastereoselective organocatalytic synthesis of (*R*)- and (*S*)-methoxycarbonylhydroxymethyluridines (mchm^5^Us) and their acid analogues (chm^5^Us) was developed. The method employs organo- and organometallic-catalyzed cyanosilylation of 5-formyluridine to yield diastereomerically enriched TMS-protected cyanohydrins, which are converted *via* a Pinner reaction to (*R*)- and (*S*)-mchm^5^Us, then hydrolyzed to (*R*)- and (*S*)-chm^5^Us.

## Abstract 2

First diastereoselective synthesis of biologically relevant (*R*)- and (*S*)- methoxycarbonylhydroxymethyluridines (mchm^5^Us) and their acid analogues (chm^5^Us) was developed.

## Graphical abstract

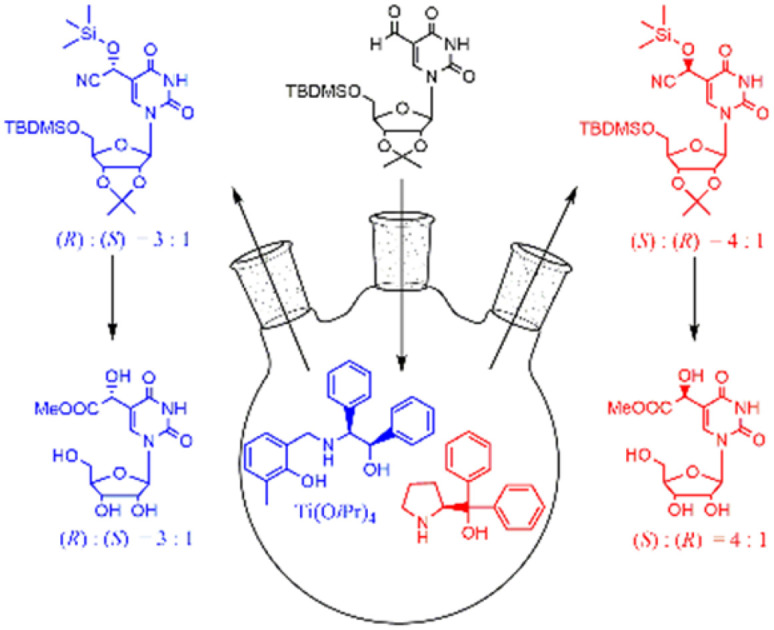

## Introduction

More than 120 modified nucleosides have been identified in transfer ribonucleic acids (tRNA), with the greatest diversity found at position 34 (the wobble position, referring to the first anticodon letter) and position 37 (adjacent to the anticodon at the 3′ end).^1^ The wobble modifications are known to influence the anticodon-codon minihelix interactions, thereby affecting the rate and fidelity of decoding process.^2,3^

**Fig. 1:**
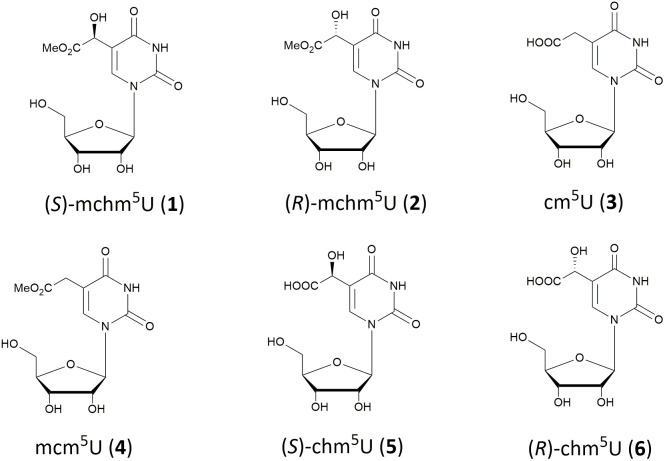
Chemical structures of (*S*)-5-methoxycarbonylhydroxymethyluridine ((*S*)-mchm^5^U, 1), (*R*)-5-methoxycarbonylhydroxymethyluridine ((*R*)-mchm^5^U, 2), 5-carboxymethyluridine (cm^5^U, 3), 5-methoxycarbonylmethyluridine (mcm^5^U, 4), (*S*)-5-carboxyhydroxymethyluridine ((*S*)-chm^5^U, 5) and (*R*)-5-carboxyhydroxymethyluridine ((*R*)-chm^5^U, 6).

A unique example of a wobble nucleoside is represented by a diastereomeric pair of 5-substituted uridine, (*S*)- and (*R*)-5-methoxycarbonylhydroxymethyluridines (abbreviated as (*S*)-mchm^5^U (1), (*R*)-mchm^5^U (2), Fig. 1). The (*S*)-isomer 1 occupies the wobble position of tRNAs^Gly^(UCC) in mammals, insects (*e.g. B. mori*), worms (*e.g. C. elegans*) and plants (*e.g. A. thaliana*).^4–8^ The (*R*)-mchm^5^U (2) is less abundant in nature and has been identified in mammalian tRNAs^Arg^(UCG).^8,9^

The biosynthetic pathway leading to the installation of the mchm group at the C5 position of uridine has been well characterized in mammalian (*S*)-mchm^5^U_34_-tRNA. The first step depends on the Elongator complex, which is composed of six Elp proteins. Elongator catalyzes the formation of 5-carboxymethyluridine (cm^5^U, 3), which is subsequently converted into 5-methoxycarbonylmethyluridine (mcm^5^U, 4) through ALKBH8-mediated methyl transfer reaction. Finally, the oxygenase domain of the same ALKBH8 enzyme catalyzes the stereoselective hydroxylation of mcm^5^U, yielding (*S*)-mchm^5^U (1).^8–10^ It has been shown that hydroxylation of mcm^5^U enhances the affinity of (*S*)-mchm^5^U_34_-tRNA^Gly^ to both GGA and GGG codons.^5^ In the absence of the mcm^5^U_34_-tRNA substrate, the oxygenase domain of ALKBH8 catalyzes hydroxylation of cm^5^U_34_, albeit inefficiently, resulting in the formation of (*S*)-5-carboxyhydroxymethyluridine ((*S*)-chm^5^U, 5).

Disturbances in mchm^5^U_34_- and chm^5^U_34_-modifying enzymes are associated with various human diseases and disabilities.^11,12^ Elp subunits are upregulated in human melanoma,^13^ breast^14^ and colon cancers;^15^ ALKBH8 is overexpressed in human urothelial carcinomas.^16^*In vivo* silencing of ALKBH8 significantly suppressed angiogenesis and growth of bladder cancers.^17^ The absence of (*S*)-mchm^5^U, (*R*)-mchm^5^U and other mcm^5^-containing uridines caused by mutational events in the human ALKBH8 gene were associated with intellectual disability and global developmental delay.^18^ In ALKBH8-KO mice, developmental abnormalities were observed, including reduced body size, impaired translation efficiency, defective erythrocyte differentiation, and loss of the wobble mcm^5^U modification.^19^ Reduced level of chm^5^U was correlated with increased resistance to platinum-based drugs in ovarian cancer patients.^20^ Notably, ALKBH8 expression – the enzyme responsible for installing chm^5^U – was significantly lower in patients who experienced recurrence, suggesting that ALKBH8 enzyme may serve as a predictive biomarker for chemotherapy response.^20^

The biological functions of the diastereomeric wobble modifications mchm^5^U and chm^5^U, including their roles in human diseases, still remain to be elucidated. Diastereomerically pure modifications serve as essential tools in analytical studies, enabling precise identification and quantification in biological samples, localization within RNA molecules and comparative profiling across physiological and pathological conditions. To data, no stereoselective synthetic methods for (*S*)- or (*R*)-mchm^5^Us (1, 2) have been reported.^4,5,21–23^ In this study, we present a pioneering approach to the stereoselective synthesis of (*S*)- and (*R*)-mchm^5^U isomers (1, 2) *via* organo- and organometallic-catalyzed conversion of 5-formyluridine into diastereoenriched cyanohydrins, followed by hydrolysis of the intermediate Pinner salts. In addition, (*S*)- and (*R*)-chm^5^U (5, 6) were synthesized by acidic hydrolysis of stereochemically pure mchm^5^U esters (1, 2).

## Results and discussion

In our previous work, a silyl-protected cyanohydrin of 5-formyluridine was successfully synthesized and converted into a racemic mixture of (*S*)- and (*R*)-mchm^5^U isomers.^21^ Since the asymmetric cyanation of aldehydes and ketones is a highly versatile method for producing stereochemically pure cyanohydrins,^24^ we aimed to develop an organocatalyzed system to promote the stereoselective cyanation of 5-formyluridine. According to the literature, the most commonly used conditions involve trimethylsilyl cyanide (TMSCN) as the cyanide source and a titanium(iv)-based catalyst in dichlomethane (DCM) as the solvent. Trimethylsilyl cyanide prevents the reversibility of the cyanation reaction by forming a trimethylsilyl ether protection (–C(CN)OTMS). Titanium(iv), in turn, is well established as a Lewis acid in the asymmetric synthesis of structurally diverse cyanohydrins.^24^ Considering the use of the non-polar DCM solvent in cyanosilylation, our initial focus was on modifying the 5-formyluridine substrate to increase its hydrophobicity and thus its solubility in DCM.

**Scheme 1:**
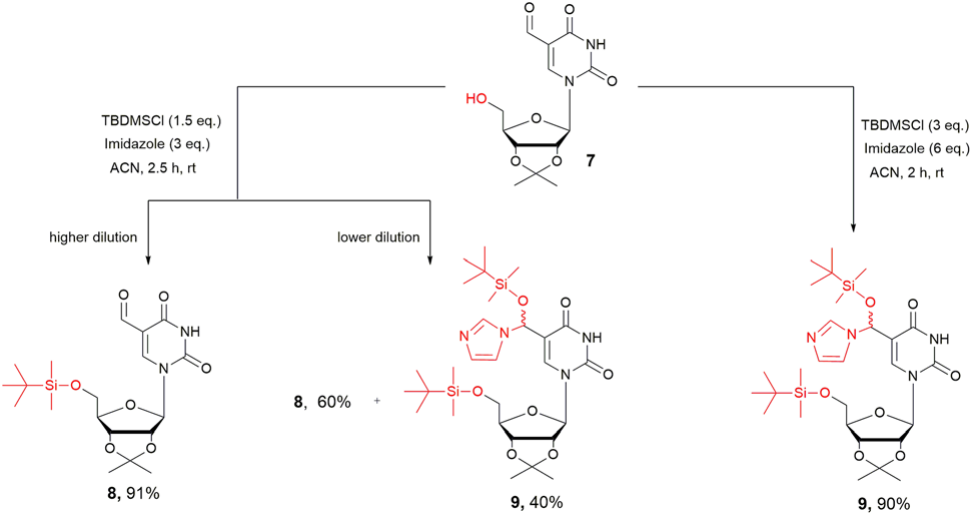
Three different pathways for the protection of 5-formyluridine (f^5^U, 7) using *tert*-butyldimethylsilyl chloride (TBDMSCl) and 1*H*-imidazole, depending on the reaction conditions (especially concentration and stoichiometry).

Readily accessible 5-formyl-2′,3′-*O*-isopropylideneuridine^21,25^ (7, Scheme 1) was employed as the starting material for subsequent protection of the 5′-hydroxyl group. The *tert*-butyldimethylsilyl (TBDMS) group was chosen with the expactation that both silyl ethers (5′-*O*-TBDMS and TMS-cyanohydrin) would be cleaved under the acidic conditions of the subsequent Pinner reaction, along with the 2′,3′-*O*-isopropylidene acetal. Therefore, 5-formyluridine 7 was treated with TBDMSCl and imidazole as an activator in acetonitrile (ACN), affording 5′-*O-tert*-butyldimethylsilyl-5-formyluridine 8 in 91% yield. A key outcome of this reaction is the complete prevention of 5-(*tert*-butyldimethylsilyloxy)-(1*H*-imidazol-1-yl)methyluridine 9 formation.^26^

The selectivity of the 5′-*O*-sililation was achieved by 2-folded increase in dilution of the reaction mixture, wherein ACN solvent was found to slightly improve the yield compared to the commonly used pyridine. Of note, when a two-fold excess of imidazole and TBDMSCl over the standard protocol was used, imidazolyl-uridine 9 was preferentially formed regardless of the reaction dilution (ESI†).

We then investigated the diastereoselective addition of TMSCN to 5-formyluridine 8, both in the presence or absence of titanium tetraisopropoxide [Ti(OiPr)_4_], using a variety of chiral ligands, including β-aminoalcohols (A, B), diphenylprolinoles (C, D), 1,1′-bis(2-naphtol) (E), tartaric acid ester (F), quinine (G), squaramides (H–J) and a thiourea derivative (K) (Table 1). To the best of our knowledge, ligands C–E and G–J have not been previously reported for use in asymmetric cyanohydrin synthesis.

**Table 1:** Optimization of the cyanosilylation reaction

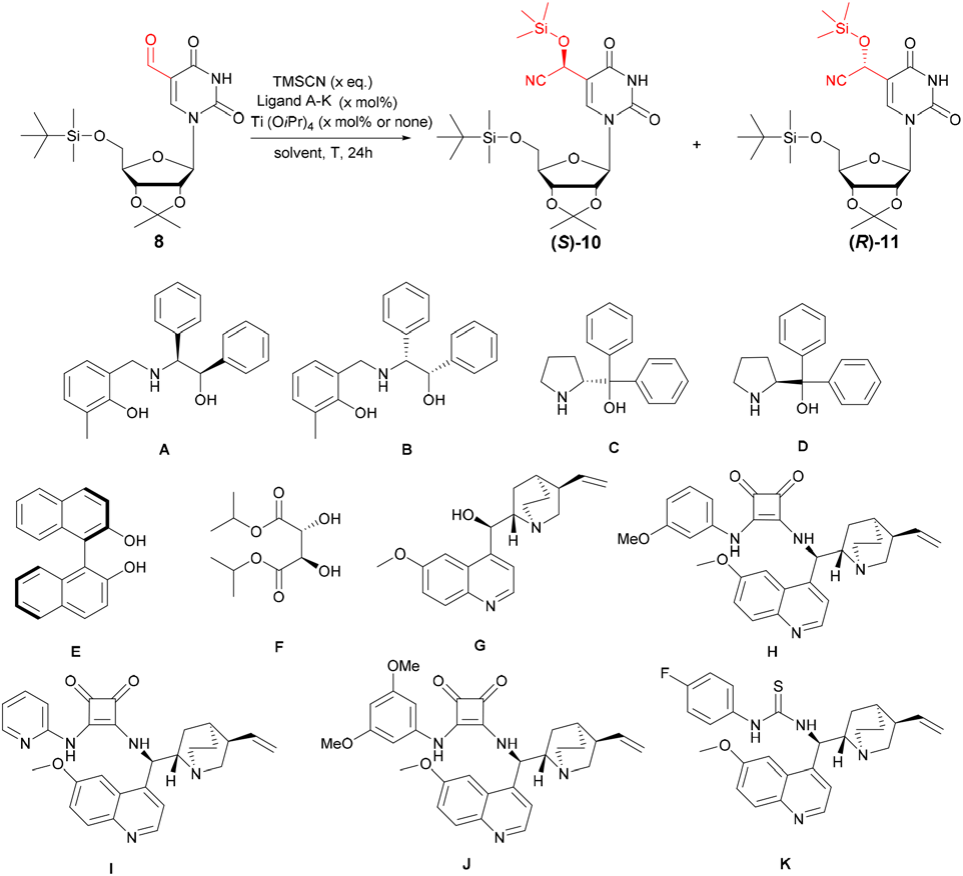					
Entry	Ligand		Ti(OiPr)_4_*x* mol%	TMSCN *x* eq.	dr (*S*) : (*R*) 10 : 11^f^
	Type	*x* mol%			
**Catalyst system selection**					
**1** a	A	**20**	**20**	**2**	**1 : 3**
2^a^	B	20	20	2	1 : 1
3^a^	C	40	20	2	1 : 1
4^a^	D	40	20	2	3 : 1
5^a^	E	20	20	2	1 : 1
6^a^	F	20	20	2	1 : 2
7^a^	G	40	20	2	2 : 1
8^a^	A	20	—	2	1 : 2
**9** a	D	**20**	**—**	**2**	**4 : 1**
10^a^	H	20	—	2	1 : 2,5
11^a^	I	20	—	2	1,5 : 1
12^a^	J	20	—	2	1 : 2,5
13^a^	K	20	—	2	1 : 1,5

**Optimization of the cyanosilylation reaction using selected ligands**A**and**D					
14^a^	A	20	20	3	1 : 3
15^a^	D	20	—	3	4 : 1
16^a^	A	5	5	2	—
17^a^	A	50	50	2	1 : 3
18^a^	D	5	—	2	—
19^a^	D	50	—	2	2,5 : 1
20^b^	A	20	20	2	—
21^b^	D	20	—	2	—
22^c^	A	20	20	2	1 : 2,5
23^c^	D	20	—	2	2,5 : 1
24^d^	A	20	20	2	1 : 1
25^d^	D	20	—	2	2 : 1
26^e^	A	20	20	2	—
27^e^	D	20	—	2	2 : 1

a Reaction was carried out under argon in DCM at −20 °C.

b Reaction was carried out in DCM at −78 °C.

c Reaction was carried out in DCM at room temperature.

d Reaction was carried out in ACN at −20 °C.

e Reagents were added into the reaction in reverse order (TMSCN first, then substrate 8) in DCM at −20 °C.

f Diastereomeric ratio (dr) was estimated based on the ^1^H NMR spectrum.

In our initial studies, we focused on selecting a catalyst system that enhances the stereoselectivity of f^5^U cyanosilylation (Table 1). All reactions were carried out with 2 equivalents (eq.) of TMSCN in DCM at −20 °C for 24 h. The first set of reactions employed an *in situ* generated titanium-based catalyst system consisting of 20 mol% Ti(OiPr)_4_ and either 20 or 40 mol% of the ligand (entries 1–7), with the higher ligand loading used for those containing only a single hydroxyl group for coordination with Ti(iv). The Ti(iv)-ligand A system (entry 1) demonstrated significant potential, affording a diastereomeric ratio of (*S*) : (*R*) = 1 : 3. Its enantiomeric counterpart, ligand B, afforded the same ratio of both diastereomers (entry 2). β-Aminoalcohol ligands A and B have previously been utilized in the asymmetric synthesis of cyanohydrins from aldehydes.^27^ In turn, the use of enantiomerically pure (*S*)-diphenyl(pyrrolidin-2-yl)methanol (D), not yet studied in cyanohydrin synthesis, afforded diastereoenriched cyanohydrins with an (*S*) : (*R*) ratio of 3 : 1 (entry 4). Next, promising ligands A and D, along with squaramide (H–J) and thiourea (K) ligands, were tested in cyanosilylation reactions without Ti(OiPr)_4_ (entries 8–13). Ligand D showed a notable improvement in stereoselectivity, achieving a (*S*) : (*R*) ratio of 4 : 1 (entry 9). Catalysts H–K also enhanced the stereoselectivity of f^5^U (7) cyanosilylation, albeit at lower diastereomeric ratios (entries 10–13).

In the subsequent step, cyanosilylation was optimized using solely ligands A and D in terms of reagent ratios (TMSCN, entries 14 and 15; ligands and Ti(OiPr)_4_, entries 16–19), reaction temperature (−78 °C or room temperature, entries 20–23), solvent (ACN, entries 24 and 25), and the order of reagent addition (entries 26 and 27). However, none of these modifications improved the diastereomeric ratio.

Overall, the optimal conditions for diasteroselective synthesis of (*R*)-cyanohydrin 11 involved the use of 20 mol% of ligand A and 20 mol% of Ti(OiPr)_4_ (−20 °C, 24 h, entry 1). In turn, the highest diastereoselectivity of (*S*)-cyanohydrin 10 formation was achieved in organocatalytic reaction with ligand D (−20 °C, 24 h, entry 9).

**Scheme 2:**
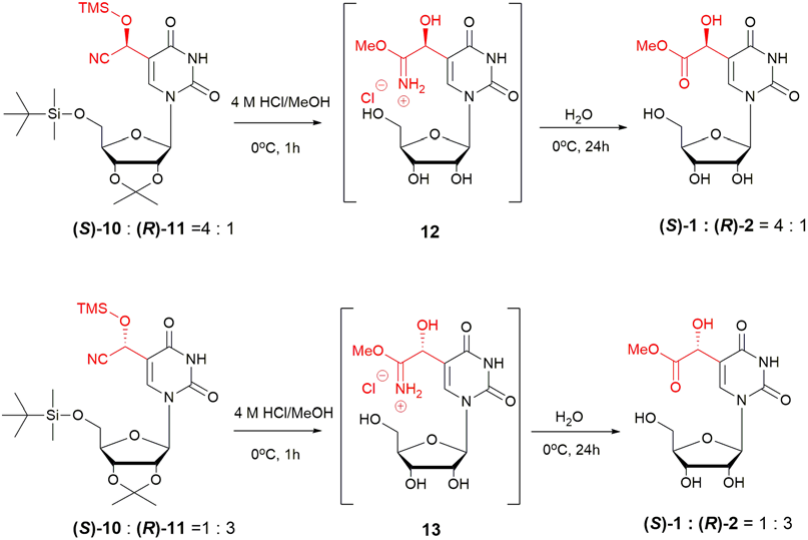
Preparation of (*S*)-mchm^5^U 1 and (*R*)-mchm^5^U 2.

The silyl-protected cyanohydrins 10 and 11 proved unstable during chromatographic purification. Therefore, the crude diastereoenriched cyanohydrins with major (*S*)-10 or (*R*)-11 isomer (Scheme 2) were subjected to the Pinner reaction under previously established conditions.^21^ Treatment with 4 M HCl in methanol furnished the corresponding imidate salts (called also the Pinner salts) enriched in (*S*)-12 or (*R*)-13 isomer, respectively. Residual hydrogen chloride was carefully removed to prevent ester hydrolysis. Subsequent incubation of the imidates in freezing water overnight afforded the corresponding esters with major (*S*)-1 or (*R*)-2 diastereomer, respectively.

The diastereoenriched esters were purified by column chromatography in ∼80% yield (3 steps) and analysed by NMR (ESI†), confirming that the (*S*) : (*R*) ratio remained unchanged from the parent cyanohydrins. Preparative RP-HPLC was then used to isolate diastereomerically pure esters yielding 60% of (*S*)-1 and 15% of (*R*)-2 from the 4 : 1 (*S*) : (*R*) mixture, and 18% of (*S*)-1 and 54% of (*R*)-2 from the 1 : 3 mixture.

**Scheme 3:**
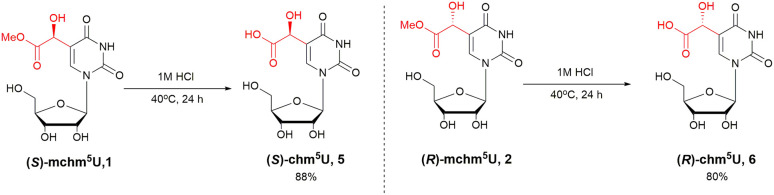
Synthesis of (*S*)-chm^5^U (5) and (*R*)-chm^5^U (6).

A scaled-up cyanosilylation reaction proceeded smoothly, demonstrating the practical utility of this method for the synthesis of mchm^5^U-based compounds, *e.g.* the as-yet-unpublished (*S*)- and (*R*)-mchm^5^U phosphoramidite monomeric units. In the final step, pure (*S*)-mchm^5^U (1) and (*R*)-mchm^5^U (2) were converted into the corresponding acids, (*S*)-chm^5^U (5) and (*R*)-chm^5^U (6), respectively (Scheme 3). To avoid racemization, the ester hydrolysis was optimized using aqueous (aq.) HCl by adjusting the reaction time, acid concentration and temperature. Under optimal conditions – 1 M aq HCl at 40 °C for 24 hours – (*S*)-chm^5^U (5) and (*R*)-chm^5^U (6) were obtained in 88% and 80% yield, respectively, following preparative RP HPLC purification. Chromatographic and NMR spectroscopic analyses (ESI†) confirmed the stereochemical integrity of both products, with no detectable racemization.

The stereochemical assignments of (*S*)- and (*R*)-mchm^5^U (1 and 2) and (*S*)- and (*R*)-chm^5^U (5 and 6) were confirmed by comparison of their circular dichroism (CD) spectra with previously reported data (ESI†).^9,21,22^

## Conclusions

In summary, we have developed an organo- and organometallic catalytic systems for the diastereoselective cyanosililation of 5-formyluridine 8, enabling the synthesis of (*S*)- and (*R*)-methoxycarbonylhydroxymethyluridines (1 and 2). The resulting esters were efficiently hydrolyzed to the corresponding acids, (*S*)-chm^5^U (5) and (*R*)-chm^5^U (6) under racemization-free conditions. Among the catalytic systems evaluated, diphenylprolinoles (C, D), 1,1′-bis(2-naphtol) (E), quinine (G), squaramides (H–J) and thiourea (K) ligands were explored for the first time in the cyanosililation of aldehydes. Optimal diastereoselective synthesis of the (*S*)-cyanohydrin ((*S*) : (*R*) = 4 : 1) was achieved using ligand D, whereas the (*R*)-cyanohydrin ((*S*) : (*R*) = 1 : 3) was obtained using ligand A in combination with Ti(OiPr)_4_ catalyst. Major goal for future research includes the convertion of (*S*)- and (*R*)-mchm^5^U esters (1 and 2) into diastereomerically pure phosphoramidites, enabling their incorporation into the wobble position of tRNA anticodon stem-loop (ASL) oligomers and subsequent evaluation of the functional roles of wobble uridine modifications.

## Experimental

### General information

All commercially available reagents were used without further purification. Catalysts were either commercially available or prepared according to the literature procedures.^27^ Solvents were freshly distilled and store over 4 Å molecular sieves prior to use. Obtained products were purified by column chromatography on silica gel (high-purity grade, pore size 60 Å, 230–400 mesh particle size, 40–63 μm particle size).

NMR spectra were registered at 400 MHz and 700 MHz (^1^H NMR), 100 MHz and 176 MHz (^13^C NMR) in D_2_O or CDCl_3_. Chemical shifts are reported in ppm relative to the deuterated solvent signal. Diastereomeric mixtures of (*R*)-5-methoxycarbonylhydroxymethyluridine ((*R*)-mchm^5^U, 1) and (*S*)-5-methoxycarbonylhydroxymethyluridine ((*S*)-mchm^5^U, 2) or (*R*)- and (*S*)-5-carboxyhydroxymethyluridine (5 and 6) were purified using Waters 515 HPLC system equipped with a 996 spectral diode array detector (column: Ascentis® C18, 100 Å, 10 μm, 25 cm × 21.2 mm; eluent: water). HPLC analysis were performed using Shimadzu Prominence HPLC system equipped with a SPD-M20A spectral photodiode array detector (column: Kinetex® C18, 100 Å, 5 μm, 250 mm × 4.6 mm; eluent: linear gradient of water and acetonitrile or linear gradient of 0.1 M CH_3_COONH_4_ and acetonitrile). HRMS spectra were recorded using electron spray ionization time-of-flight (ESI-TOF) spectrometry. The measurement was performed in positive ion mode with capillary voltage set to 4.5 kV. CD spectra were performed on Jasco J-1500 spectrophotometer using quartz cell with a 0.1 cm path length. The measurements were recorded at 21 °C in the wavelength range from 190 to 350 nm with a 5 nm data point interval. The buffer spectrum was subtracted from the sample spectra, and the resultant CD spectra were smoothed with a Savitzky–Golay algorithm (5 convolution coefficient).

### Synthesis of 5′-*O-tert*-butyldimethylsilyl-5-formyl-2′,3′-*O*-isopropylideneuridine (8)

5-Formyl-2′,3′-*O*-isopropylideneuridine (7) (3.00 g, 9.6 mmol) was dissolved in freshly distilled acetonitrile (121 mL) and 1*H-*imidazole (1.96 g, 28.8 mmol) was added. The solution was stirred for 15 minutes at room temperature. *Tert*-butyldimethylsilyl chloride (2,17 g, 14.4 mmol) was then added and the reaction mixture was stirred for an additional 2.5 hours at room temperature. The solvent was removed *in vacuo* and crude product was purified by column chromatography using DCM : acetone (50/1 v/v) solvent system. Compound 8 was obtained in 91% yield (3.73 g). ^1^H NMR (400 MHz, Chloroform-*d*) *δ* 0.02 (s, 3H), 0.07 (s, 3H), 0.84 (s, 9H), 1.37 (s, 3H), 1.60 (s, 3H), 3.80 (dd, *J* = 11.8, 2.7 Hz, 1H), 3.98 (dd, *J* = 11.8, 2.1 Hz, 1H), 4.55 (q, *J* = 2.1 Hz, 1H), 4.73 (dd, *J* = 6.0, 1.6 Hz, 1H), 4.78 (dd, *J* = 6.0, 2.6 Hz, 1H), 5.81 (d, *J* = 2.6 Hz, 1H), 8.43 (s, 1H), 10.00 (s, 1H). ^13^C NMR (176 MHz, Chloroform-*d*) *δ* −5.55, −5.28, 18.47, 25.25, 25.99, 27.24, 63.85, 81.51, 86.33, 88.29, 95.51, 110.59, 113.96, 145.93, 149.27, 161.85, 185.93. HRMS calcd for C_19_H_30_N_2_O_7_Si [M + H]^+^ 427.18948, found 427.1890.

### Synthesis of (1*R*, 2*S*)-2-(*N*-2′-hydroxyl-3′-methylbenzyl)amino-1,2-diphenyl-1-ethanol (ligand A)

A mixture of (1*R*,2*S*)-2-amino-1,2-diphenylethanol (214 mg, 1.0 mmol) and 2-hydroxy-3-methylbenzaldehyde (120 μL, 1.0 mmol) in anhydrous ethanol (5 mL) was stirred at room temperature for 36 hours. Sodium borohydride (114 mg, 3.0 mmol) was then added, and the reaction mixture was stirred for an additional 2 hours. The reaction was quenched with water (5 mL) and extracted three times with DCM (3 × 20 mL). The combined organic layers were washed with brine and dried over anhydrous sodium sulphate. The drying agent was filtered and solvents were removed under reduced pressure. The product was purified by silica gel column chromatography using a DCM : acetone solvent system in a 50 : 1 volume ratio. (1*R*, 2*S*)-2-(*N*-2′-hydroxyl-3′-methylbenzyl)amino-1,2-diphenyl-1-ethanol (ligand A) was obtained in 72% yield (240 mg). ^1^H NMR (700 MHz, Chloroform-*d*) *δ* 2.22 (s, 3H), 3.53 (d, *J* = 13.7 Hz, 1H), 3.78 (d, *J* = 13.7 Hz, 1H), 3.86 (d, *J* = 6.2 Hz, 1H), 4.88 (d, *J* = 6.1 Hz, 1H), 6.58–6.67 (m, 2H), 7.00 (dd, *J* = 7.1, 2.0 Hz, 1H), 7.26 (s, 10H). ^13^C NMR (176 MHz, Chloroform-*d*) *δ* 15.83, 50.41, 68.36, 77.58, 118.73, 122.02, 125.31, 126.08, 126.91 (2 × C), 128.18, 128.30, 128.50 (2 × C), 128.53 (2 × C), 128.68 (2 × C), 130.02, 137.77, 140.48, 156.01. HRMS calcd for C_22_H_23_NO_2_ [M + H]^+^ 334.18014, found 334.1804.

### Synthesis of (1*S*,2*R*)-2-(*N*-2′-hydroxyl-3′-methylbenzyl)amino-1,2-diphenyl-1-ethanol (ligand B)

A mixture of (1*S*,2*R*)-2-amino-1,2-diphenylethanol (214 mg, 1.0 mmol) and 2-hydroxy-3-methylbenzaldehyde (120 μL, 1.0 mmol) in anhydrous ethanol (5 mL) was stirred at room temperature for 36 hours. Sodium borohydride (114 mg, 3.0 mmol) was then added, and the reaction mixture was stirred for an additional 2 hours. The reaction was quenched with water (5 mL) and extracted three times with DCM (3 × 20 mL). The combined organic layers were washed with brine and dried over anhydrous sodium sulphate. The drying agent was filtered and solvents were removed under reduced pressure. The product was purified by silica gel column chromatography using a DCM : acetone solvent system in a 50 : 1 volume ratio. (1*S*, 2*R*)-2-(*N*-2′-hydroxyl-3′-methylbenzyl)amino-1,2-diphenyl-1-ethanol (ligand B) was obtained in 75% yield (250 mg). ^1^H NMR (700 MHz, Chloroform-*d*) *δ* 2.23 (s, 3H), 3.56 (d, *J* = 13.7 Hz, 1H), 3.82 (d, *J* = 13.6 Hz, 1H), 3.90 (d, *J* = 6.3 Hz, 1H), 4.94 (d, *J* = 6.2 Hz, 1H), 6.55–6.69 (m, 2H), 6.98–7.03 (m, 1H), 7.26 (s, 10H).^13^C NMR (176 MHz, Chloroform-*d*) *δ* 15.87, 50.47, 68.58, 77.70, 118.83, 122.00, 125.45, 126.19, 126.97 (2 × C), 128.37, 128.47, 128.61 (2 × C), 128.65 (2 × C), 128.83 (2 × C), 130.14, 137.68, 140.44, 156.02. HRMS calcd for C_22_H_23_NO_2_ [M + H]^+^ 334.18014, found 334.1803.

### Synthesis of (*S*)-5-methoxycarbonylhydroxymethyluridine ((*S*)-mchm^5^U, 1) using ligand D

The (*S*)-diphenyl(pyrrolidin-2-yl)methanol (ligand D) (12.7 mg, 50 μmol) was dissolved in anhydrous DCM (0.3 mL) and the solution of 5′-*O-tert*-butyldimethylsilyl-5-formyl-2′,3′-*O*-isopropylideneuridine 8 (106.0 mg, 250 μmol) in 0.3 mL of dry DCM was added. The reaction mixture was stirred for 15 minutes at room temperature. After cooling to −20 °C trimethylsilylcyanide (62.6 μL, 500 μmol) was added dropwise. The reaction mixture was stirred for an additional 24 hours at −20 °C. The solvent was removed *in vacuo*. A crude mixture containing (*S*)- and (*R*)-5-(trimethylsilyloxy)cyanomethyl-2′,3′-*O*-isopropylidene-5′-*O*-trimethylsilyluridine (10 and 11) was obtained (150 mg). The diastereomeric ratio of the resulting cyanohydrins (*S*) : (*R*) = 4 : 1 was estimated by ^1^H NMR. The crude mixture of cyanohydrins 10 and 11 (40 mg) in (*S*) : (*R*) = 4 : 1 ratio was treated with 4 M HCl/MeOH at 0 °C. To generate hydrogen chloride, acetyl chloride (1.35 mL), distilled prior to use, was added dropwise to anhydrous methanol (3.4 mL) in an ice bath. The reaction progress was monitored by thin-layer chromatography (TLC) using a isopropanol : water (4 : 1, v/v) solvent system. After one hour the solvent was removed under reduced pressure and the solid residue was co-evaporated three times with methanol to remove the excess of hydrogen chloride. The flask was placed in an ice bath, and distilled water (4 mL) was added. The mixture was stirred for 24 hours at 5 °C. Diastereomers 1 and 2 were separated using preparative HPLC (Rt for (*S*) = 8.3 min; Rt for (*R*) = 10.7 min). Structure of isomers was confirmed by ^1^H and ^13^C NMR spectral analysis. The diastereomeric ratio of (*S*) : (*R*) = 4 : 1 was confirmed by the ratio of peak areas in the UV-monitored HPLC chromatogram of reaction mixture. Crude mixture of esters was purified from catalyst byproducts using flash column chromatography (eluent : chloroform : methanol, 85 : 15, v/v). Diastereomerically pure esters (*S*)-1 and (*R*)-2 were isolated in 60% and 15% yields, respectively. (*S*)-mchm^5^U (1) ^1^H NMR (700 MHz, deuterium oxide) *δ* 3.81 (s, 3H), 3.86 (dd, *J* = 12.9, 4.0 Hz, 1H), 3.98 (dd, *J* = 12.9, 2.8 Hz, 1H), 4.18 (ddd, *J* = 6.3, 4.0, 2.8 Hz, 1H), 4.28 (t, *J* = 5.6 Hz, 1H), 4.39 (dd, *J* = 5.3, 4.0 Hz, 1H), 5.15 (s, 1H), 5.96 (d, *J* = 4.0 Hz, 1H), 8.13 (d, *J* = 0.5 Hz, 1H). ^13^C NMR (176 MHz, deuterium oxide) *δ* 53.17, 60.40, 67.05, 69.10, 73.91, 84.11, 89.61, 112.39, 141.13, 151.31, 163.75, 173.66. HRMS calcd for C_12_H_16_N_2_O_9_ [M + H]^+^ 333.09284, found 333.0930.

### Synthesis of (*R*)-5-methoxycarbonylhydroxymethyluridine ((*R*)-mchm^5^U, 2) using ligand A and Ti(iv)

The catalyst ligand A (16.7 mg, 50 μmol) was dissolved in anhydrous DCM (0.3 mL) under inert conditions. After 5 minutes, titanium(iv) isopropoxide (15.3 μL, 50 μmol) was added dropwise. The flask was sealed with a stopper and placed in an oil bath at 30 °C. Reaction mixture was then transferred to an dry ice-acetone bath, and upon cooling to −20 °C, 5′-*O-tert*-butyldimethylsilyl-5-formyl-2′,3′-*O*-isopropylideneuridine 8 (106.0 mg, 250 μmol) in 0.3 mL DCM was added. The reaction mixture was stirred for an additional 10 minutes at −20 °C. Trimethylsilyl cyanide (62.6 μL, 500 μmol) was then added dropwise. The reaction mixture was stirred for an additional 24 hours at −20 °C. The solvent was removed under reduced pressure and a crude mixture of (*R*)- and (*S*)-5-(trimethylsilyloxy)cyanohydroxymethyl-2′,3′-*O*-isopropylidene-5′-*O*-trimethylsilyluridine 11 and 10 was obtained (146 mg). The diastereomeric ratio of the resulting cyanohydrins, (*R*) : (*S*) = 3 : 1 was estimated by ^1^H NMR. The crude mixture of cyanohydrins 11 and 10 (40 mg) in (*R*) : (*S*) = 3 : 1 ratio was treated with 4 M HCl/MeOH at 0 °C. To generate hydrogen chloride, acetyl chloride (1.35 mL), distilled prior to use, was added dropwise to anhydrous methanol (3.4 mL) in an ice bath. The reaction progress was monitored by TLC using a isopropanol : water (4 : 1, v/v) solvent system. After one hour the solvent was removed under reduced pressure and the solid residue was co-evaporated three times with methanol to remove the excess of hydrogen chloride. The flask was placed in an ice bath, and distilled water (4 mL) was added. The mixture was stirred for 24 hours at 5 °C. Diastereomers 2 and 1 were separated using preparative HPLC (Rt for (*S*) = 8.3 min; Rt for (*R*) = 10.7 min). Structure of isomers was confirmed by ^1^H and ^13^C NMR spectral analysis. The diastereomeric ratio of (*R*) : (*S*) = 3 : 1 was confirmed by the ratio of peak areas in the UV-monitored HPLC chromatogram in reaction mixture. Crude mixture of esters was purified from catalyst byproducts using flash column chromatography (eluent : chloroform : methanol, 85 : 15, v/v). Diastereomerically pure esters (*R*)-2 and (*S*)-1 were isolated in 54% and 18% yields, respectively. (*R*)-mchm^5^U (2). ^1^H NMR (700 MHz, deuterium oxide) *δ* 3.81 (s, 3H), 3.86 (dd, *J* = 12.9, 4.0 Hz, 1H), 3.98 (dd, *J* = 12.8, 2.8 Hz, 1H), 4.18 (ddd, *J* = 6.2, 3.9, 2.8 Hz, 1H), 4.25–4.30 (m, 1H), 4.39 (dd, *J* = 5.3, 4.0 Hz, 1H), 5.15 (s, 1H), 5.95 (d, *J* = 4.0 Hz, 1H), 8.14 (d, *J* = 0.6 Hz, 1H). ^13^C NMR (176 MHz, deuterium oxide) *δ* 53.16, 60.34, 67.04, 69.08, 73.92, 84.11, 89.64, 112.38, 141.17, 151.34, 173.66. HRMS calcd for C_12_H_16_N_2_O_9_ [M + H]^+^ 333.09284, found 333.0932.

### Synthesis of (*S*)-5-carboxyhydroxymethyluridine (5)

Aqueous solution of 1 N HCl (0.5 mL) was added to (*S*)-5-methoxycarbonylhydroxymethyluridine 1 (10.00 mg, 30 μmol). The reaction mixture was stirred for 24 hours at 40 °C. The solvent was removed under reduced pressure and the solid residue was co-evaporated three times with water to remove the excess of hydrogen chloride. The crude (*S*)-5-carboxyhydroxymethyluridine 5 was purified using RP-HPLC chromatography (water as eluent) in 88% (8.4 mg). ^1^H NMR (700 MHz, deuterium oxide) *δ* 3.85 (dd, *J* = 12.8, 4.1 Hz, 1H), 3.97 (dd, *J* = 12.9, 2.9 Hz, 1H), 4.17 (ddd, *J* = 5.9, 4.1, 2.8 Hz, 1H), 4.28 (t, *J* = 5.6 Hz, 1H), 4.39 (dd, *J* = 5.3, 4.1 Hz, 1H), 5.04 (s, 1H), 5.96 (d, *J* = 4.1 Hz, 1H), 8.08 (s, 1H). ^13^C NMR (176 MHz, deuterium oxide) *δ* 60.47, 67.29, 69.15, 73.85, 84.11, 89.55, 113.06, 140.84, 151.37, 163.84, 175.62. HRMS calcd for C_11_H_14_N_2_O_9_ [M + H]^+^ 319.07719, found 319.0773.

### Synthesis of (*R*)-5-carboxyhydroxymethyluridine (6)

Aqueous solution of 1 N HCl (0.5 mL) was added to (*R*)-5-methoxycarbonylhydroxymethyluridine 2 (10.00 mg, 30 μmol). The reaction mixture was stirred for 24 hours at 40 °C. The solvent was removed under reduced pressure and the solid residue was co-evaporated three times with water to remove the excess of hydrogen chloride. The crude (*R*)-5-carboxyhydroxymethyluridine 6 was purified using RP-HPLC chromatography (water as eluent) in 80% (7.6 mg). ^1^H NMR (700 MHz, deuterium oxide) *δ* 3.85 (dd, *J* = 12.9, 4.0 Hz, 1H), 3.97 (dd, *J* = 12.9, 2.8 Hz, 1H), 4.17 (dt, *J* = 6.3, 3.5 Hz, 1H), 4.27 (t, *J* = 5.7 Hz, 1H), 4.38 (dd, *J* = 5.3, 4.0 Hz, 1H), 5.09 (s, 1H), 5.95 (d, *J* = 4.0 Hz, 1H), 8.11 (s, 1H). ^13^C NMR (176 MHz, deuterium oxide) *δ* 60.39, 66.97, 69.11, 73.90, 84.10, 89.63, 112.79, 140.97, 151.34, 163.85, 175.22. HRMS calcd for C_11_H_14_N_2_O_9_ [M + H]^+^ 319.07719, found 319.0775.

## Author contributions

The authors confirm their contribution to the paper as follows: study conception and design: T. B., G. L., synthesis: T. B., B. K., data collection: T. B., A. D., B. K., analysis and interpretation of results: T. B., A. D., draft manuscript: T. B., G. L. All authors reviewed the results and approved the final version of the manuscript.

## Conflicts of interest

There are no conflicts to declare.

## Supplementary Material

RA-015-D5RA04760A-s001

## Data Availability

The data supporting this article have been included in the ESI.†
