# Supplementary material for: First organocatalytic, diastereoselective synthesis of tRNA wobble nucleosides: (R)- and (S)-methoxycarbonylhydroxymethyluridines (mchm5Us) and their acid analogues (chm5Us)
Source: RSC Adv. 2025 Jul 29;15(33):26943–9. doi: 10.1039/d5ra04760a (PMC12305299; doi:10.1039/d5ra04760a)
Supplement: RA-015-D5RA04760A-s001 [file RA-015-D5RA04760A-s001.pdf]

Supplementary Information (SI) for RSC Advances.
This journal is © The Royal Society of Chemistry 2025

First organocatalytic, diastereoselective synthesis of tRNA wobble nucleosides: (*R*)- and (*S*)-methoxycarbonylhydroxymethyluridines (mchm^{5}Us) and their acid analogues (chm^{5}Us)

Tomasz Bartosik,*[a] Agnieszka Dziergowska,[a] Blazej Kowalski[a] and Grazyna Leszczynska[a]

Table of contents

1. **General information** 1

2. **Synthesis of of 2',3'-*O*-isopropylidene-5'-*O*-*tert*-butyldimethylsilyl-5-(*tert*-butyldimethylsilyloxy)(1-*H*-imidazol-1-yl)methyluridine 9** 2

3. **^{1}H and ^{13}C NMR spectra** 3

4. **HPLC chromatograms of (*S*)-5-methoxycarbonylhydroxymethyluridine 1 and (*R*)-5-methoxycarbonylhydroxymethyluridine 2** 11

5. **HPLC chromatograms of (*S*)-5-carboxyhydroxymethyluridine 5 and (*R*)-5-carboxy hydroxymethyluridine 6** 13

6. **MS Spectra** 14

7. **CD Spectra** 18

8. **References** 20

1. General information

All commercially available reagents were used without further purification. Catalysts were either commercially available or prepared according to literature procedures.[1] Solvents were freshly distilled and store over 4Å molecular sieves prior to use. Obtained products were purified by column chromatography on silica gel (high-purity grade, pore size 60Å, 230-400 mesh particle size, 40-63 µm particle size).

NMR spectra were registered at 400 MHz and 700 MHz (^{1}H NMR), 100 MHz and 176 MHz (^{13}C NMR) in D_2O or $CDCl_3$. Chemical shifts are reported in ppm relative to the deuterated solvent signal.

Diastereomeric mixtures of (*R*)-5-methoxycarbonylhydroxymethyluridine ((*R*)-mchm^{5}U, **1**) and (*S*)-5-methoxycarbonylhydroxymethyluridine ((*S*)-mchm^{5}U, **2**) or (*R*)- and (*S*)-5-carboxyhydroxymethyluridine (**5** and **6**) were purified using Waters 515 HPLC system equipped with a 996 spectral diode array detector (column: Ascentis® C18, 100Å, 10 µm, 25 cm x 21.2 mm; eluent: water). HPLC analysis were performed using Shimadzu Prominence HPLC system equipped with a SPD-M20A spectral photodiode array detector (column: Kinetex® C18, 100Å, 5 µm, 250 mm x 4.6 mm; eluent: linear gradient of water and acetonitrile or linear gradient of 0.1 M CH_3COONH_4 and acetonitrile).

HRMS spectra were recorded using electron spray ionization time-of-flight (ESI-TOF) spectrometry. The measurement was performed in positive ion mode with capillary voltage set to 4.5 kV.

CD Spectra was performed on Jasco J-1500 spectrophotometer using quartz cell with a 0.1 cm path length. The measurements were recorded at 21 °C in the wavelength range from 190 to 350 nm with a 5 nm data point interval. The buffer spectrum was subtracted from the sample spectra, and the resultant CD spectra were smoothed with a Savitzky-Golay algorithm (5 convolution coefficient).

2. Synthesis of 2',3'-*O*-isopropylidene-5'-*O*-*tert*-butyldimethylsilyl-5-(*tert*-butyldimethylsilyloxy)(1-*H*-imidazol-1-yl)methyluridine 9

5-Formyl-2',3'-*O*-isopropylideneuridine (**7**) (3.00 g, 9.6 mmol) was dissolved in freshly distilled acetonitrile (74 mL) and imidazole (3.92 g, 57.6 mmol) was added. The solution was stirred for 15 minutes at room temperature. *Tert*-butyldimethylsilyl chloride (4.34 g, 28.8 mmol) was then added and the reaction mixture was stirred for an additional 2 hours at room temperature. The solvent was removed *in vacuo* and crude product was purified by column chromatography using DCM : methanol (50/1 *v/v*) solvent system. Compound **9** was obtained in 90% yield (5.26 g).

^{1}H NMR (700 MHz, Acetone-d_6) δ 0.01 (d, J = 2.9 Hz, 6H), 0.07 (d, J = 4.6 Hz, 12H), 0.20 (d, J = 6.2 Hz, 6H), 0.89 (s, 18H), 0.91 (s, 18H), 1.32 (s, 6H), 1.50 (s, 3H), 1.51 (s, 3H), 3.80-3.85 (m, 4H), 4.14-4.17 (m, 2H), 4.75-4.78 (m, 2H), 5.12-5.13 (m, 2H), 5.74 (d, J = 1.8 Hz, 1H), 5.82 (d, J = 2.4 Hz, 1H), 6.64 (d, J = 1.0 Hz, 1H), 6.64 (d, J = 1.0 Hz, 1H), 6.89 (m, 2H), 7.20 (d, J = 3.7 Hz, 2H), 7.78-7.79 (m, 2H), 7.95 (d, J = 1.0 Hz, 1H), 7.97 (d, J = 1.0 Hz, 1H).

^{13}C NMR (176 MHz, Acetone-d_6) δ -6.14, -6.03, -5.97, -5.93, -5.92, -5.89, 17.75, 17.98, 18.01, 24.58, 24.68, 25.14, 25.16, 25.38, 25.40, 25.45, 26.57, 26.61, 63.73, 63.89, 75.53, 75.56, 78.34, 81.77, 81.85, 84.15, 84.41, 87.53, 88.09, 88.58, 93.95, 95.52, 113.29, 113.47, 113.56, 113.60, 116.63, 128.66, 136.15, 136.17, 139.57, 140.88, 149.89, 161.30.

HRMS calcd for $C_{28}H_{48}N_4O_7Si_2$ [M+H]+ 609.31340, found 609.2970

3. ^{1}H and ^{13}C NMR spectra

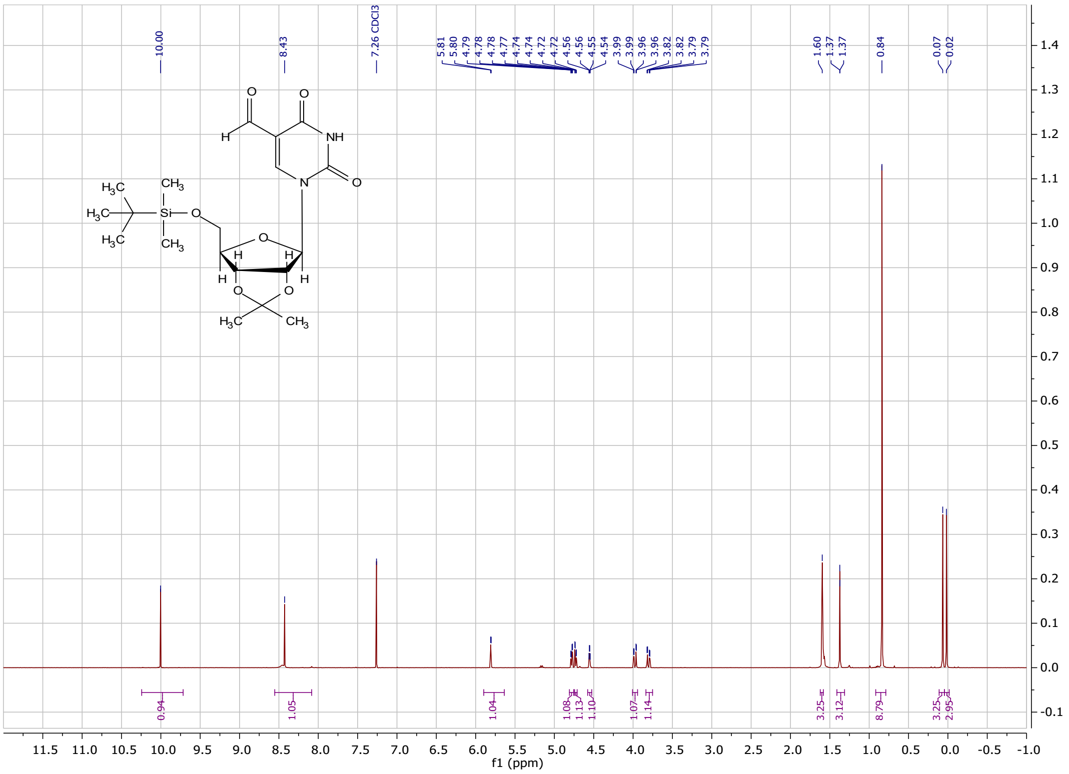

Figure S1. ^{1}H NMR spectrum of 5’-*O*-*tert*-butyldimethylsilyl-5-formyl-2’,3’-*O*-isopropylideneuridine **8**

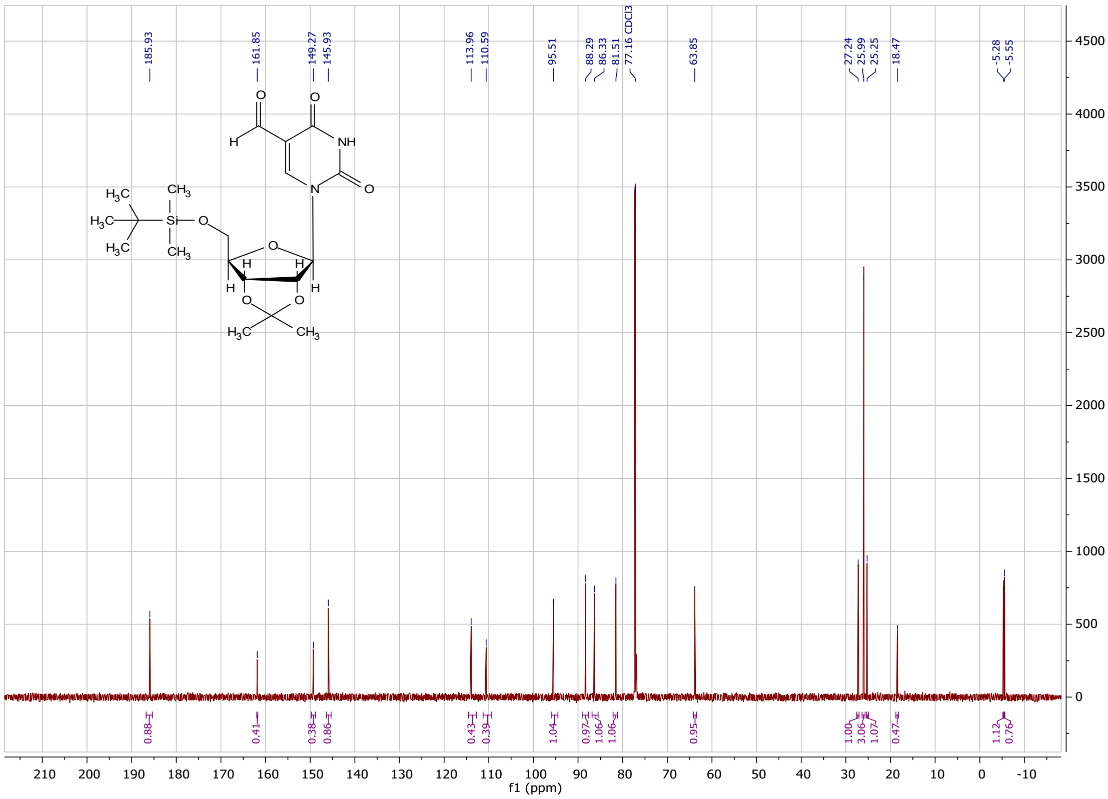

Figure S2. ^{13}C NMR spectrum of 5’-*O*-*tert*-butyldimethylsilyl-5-formyl-2’,3’-*O*-isopropylideneuridine **8**

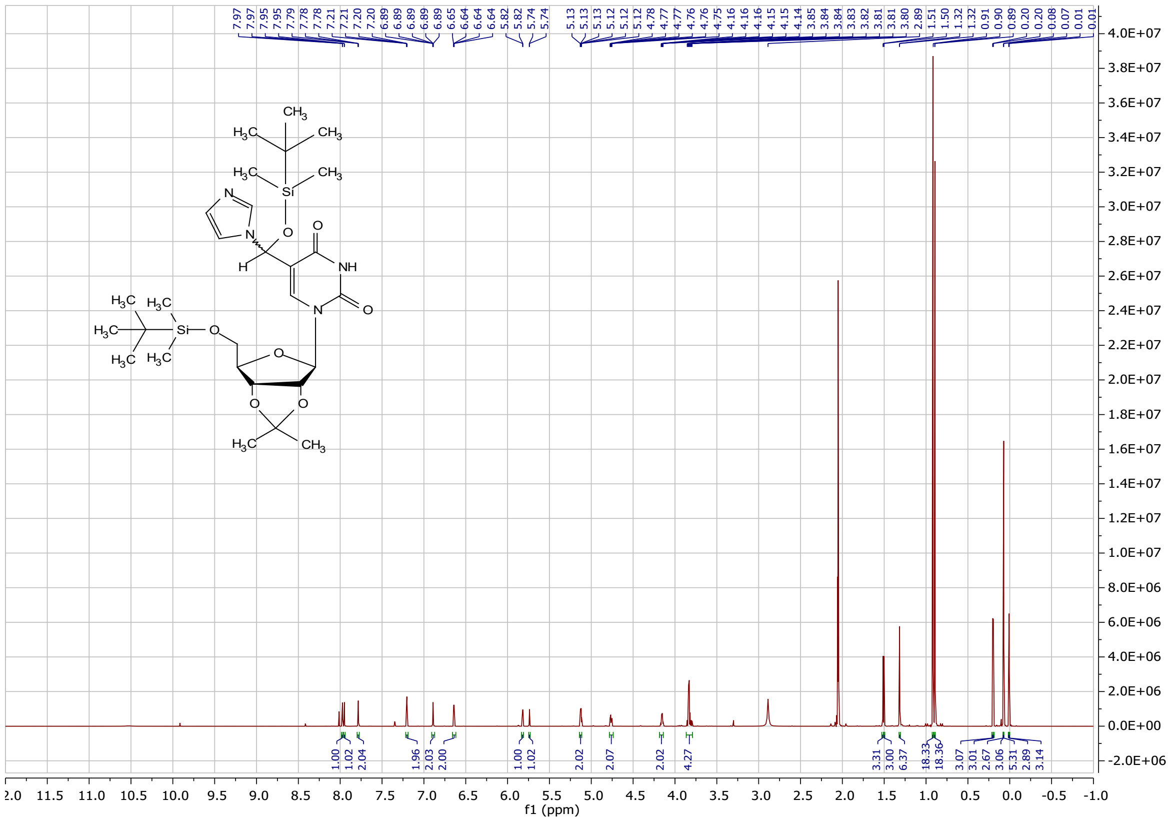

Figure S3. ^{1}H NMR spectrum of 2',3'-*O*-isopropylidene-5'-*O*-*tert*-butyldimethylsilyl-5-(*tert*-butyldimethylsilyloxy)(1-*H*-imidazol-1-yl)methyluridine **9**

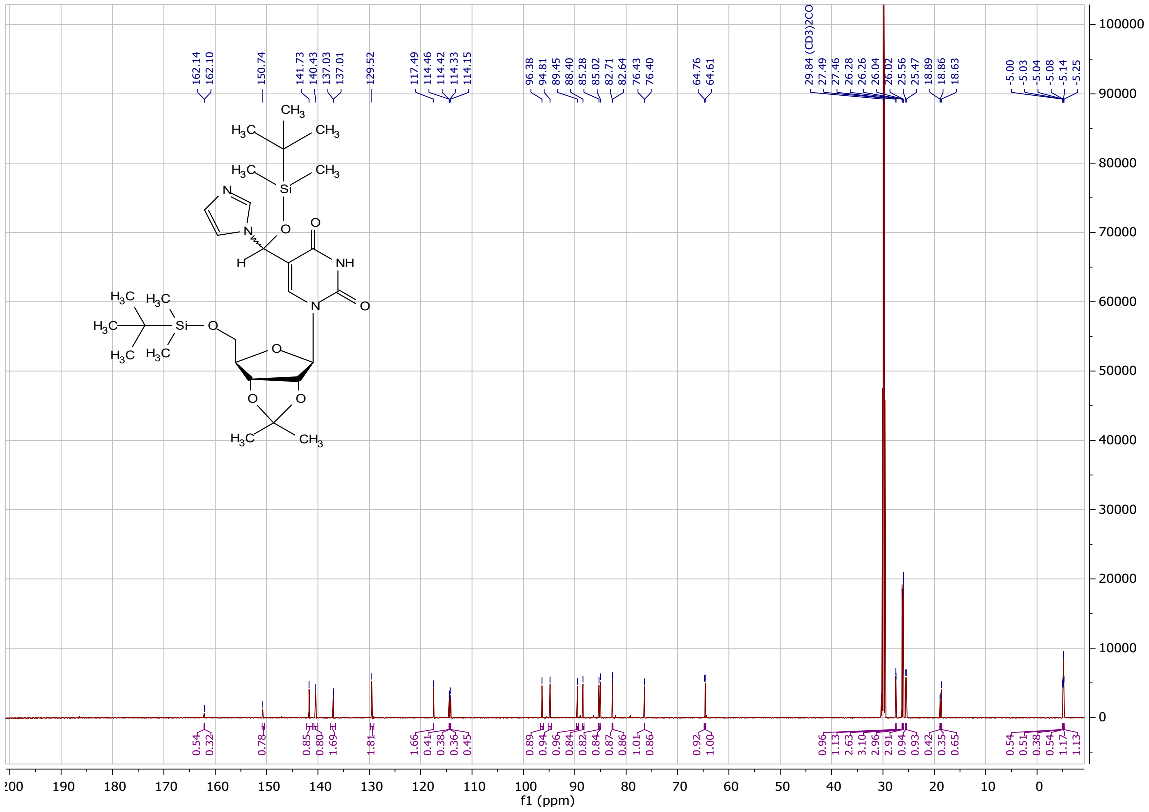

Figure S4. ^{13}C NMR spectrum of 2',3'-*O*-isopropylidene-5'-*O*-*tert*-butyldimethylsilyl-5-(*tert*-butyldimethylsilyloxy)(1-*H*-imidazol-1yl)methyluridine **9**

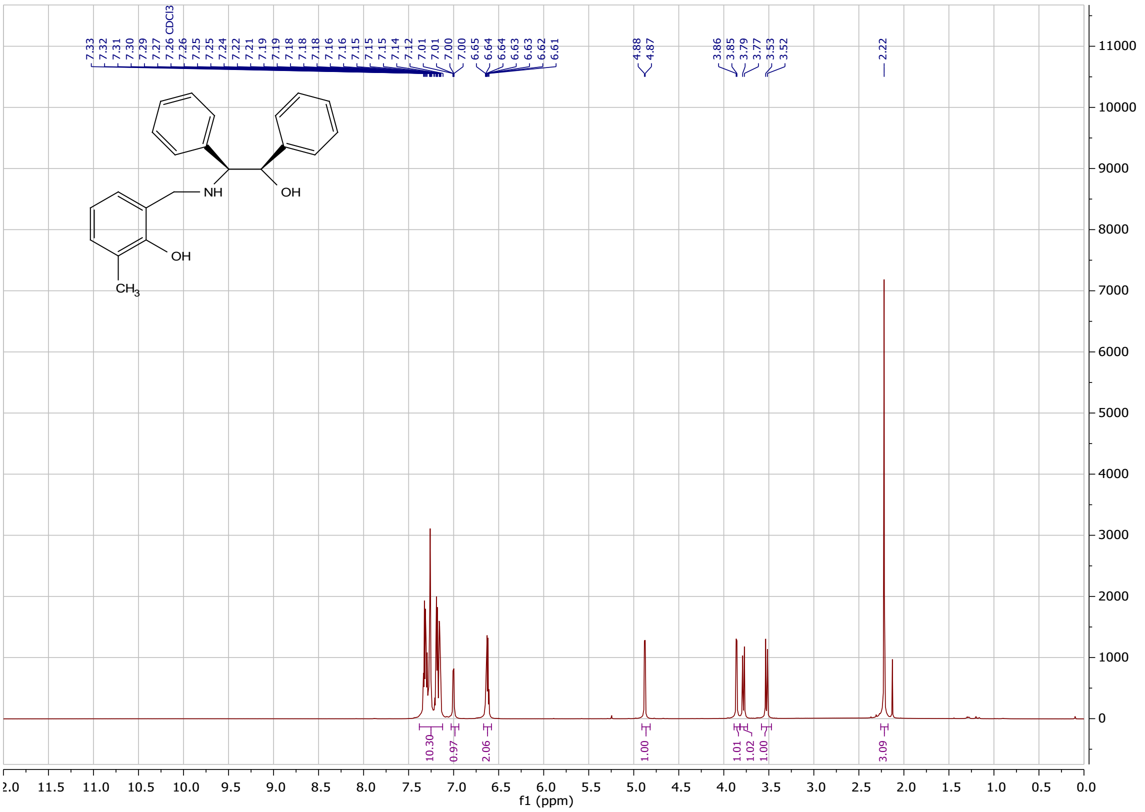

Figure S5. ^{1}H NMR spectrum of (1*R*,2*S*)-2-(*N*-2'-hydroxyl-3'-methylbenzyl)amino-1,2-diphenyl-1-ethanol (ligand **A**)

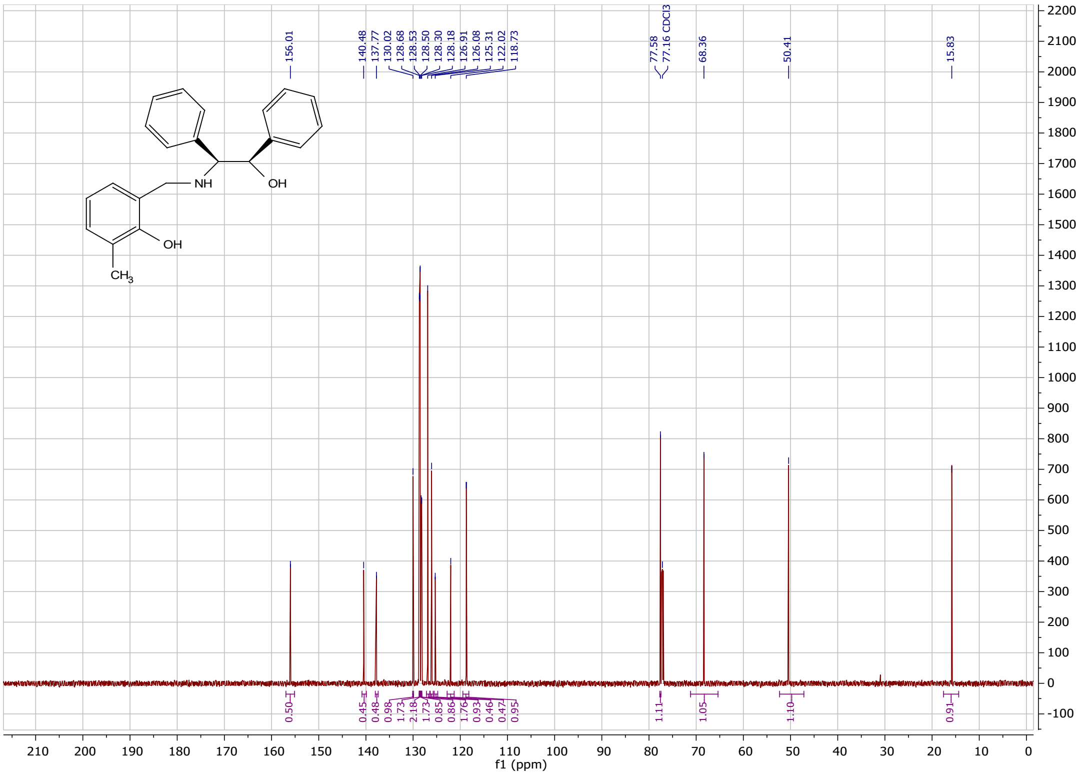

Figure S6. ^{13}C NMR spectrum of (1*R*,2*S*)-2-(*N*-2'-hydroxyl-3'-methylbenzyl)amino-1,2-diphenyl-1-ethanol (ligand **A**)

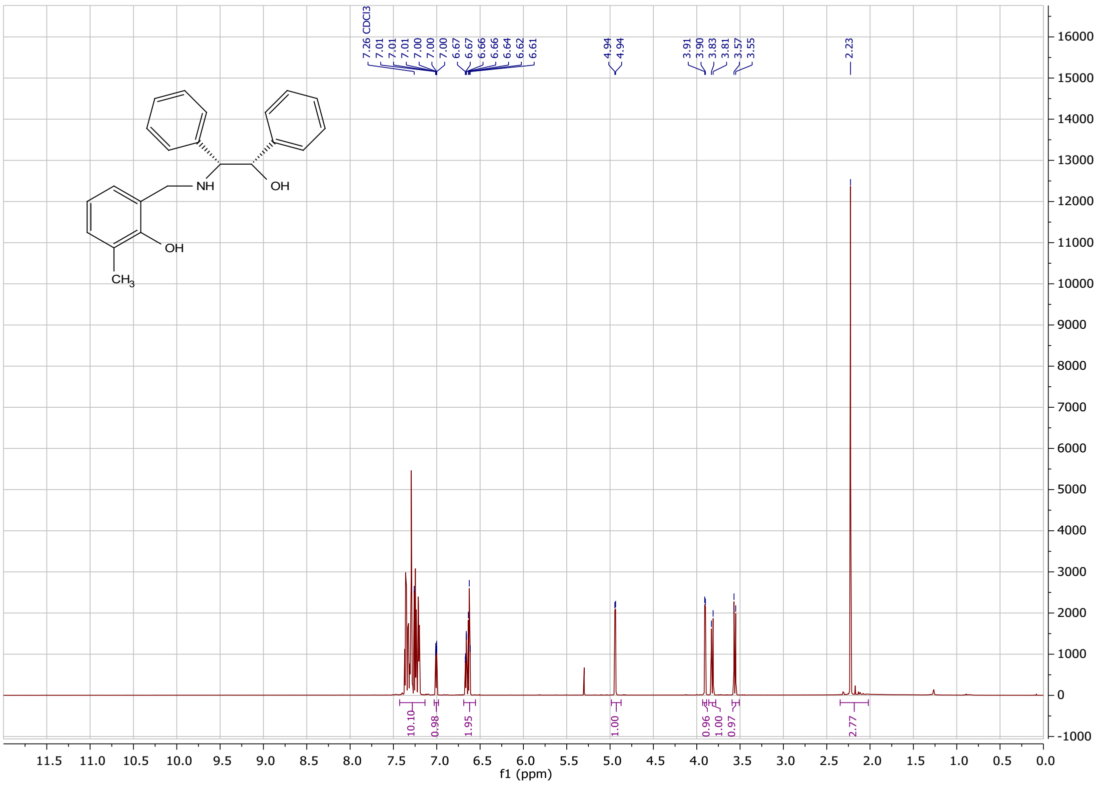

Figure S7. ^{1}H NMR spectrum of (1*S*,2*R*)-2-(N-2'-hydroxyl-3'-methylbenzyl)amino-1,2-diphenyl-1-ethanol (ligand **B**)

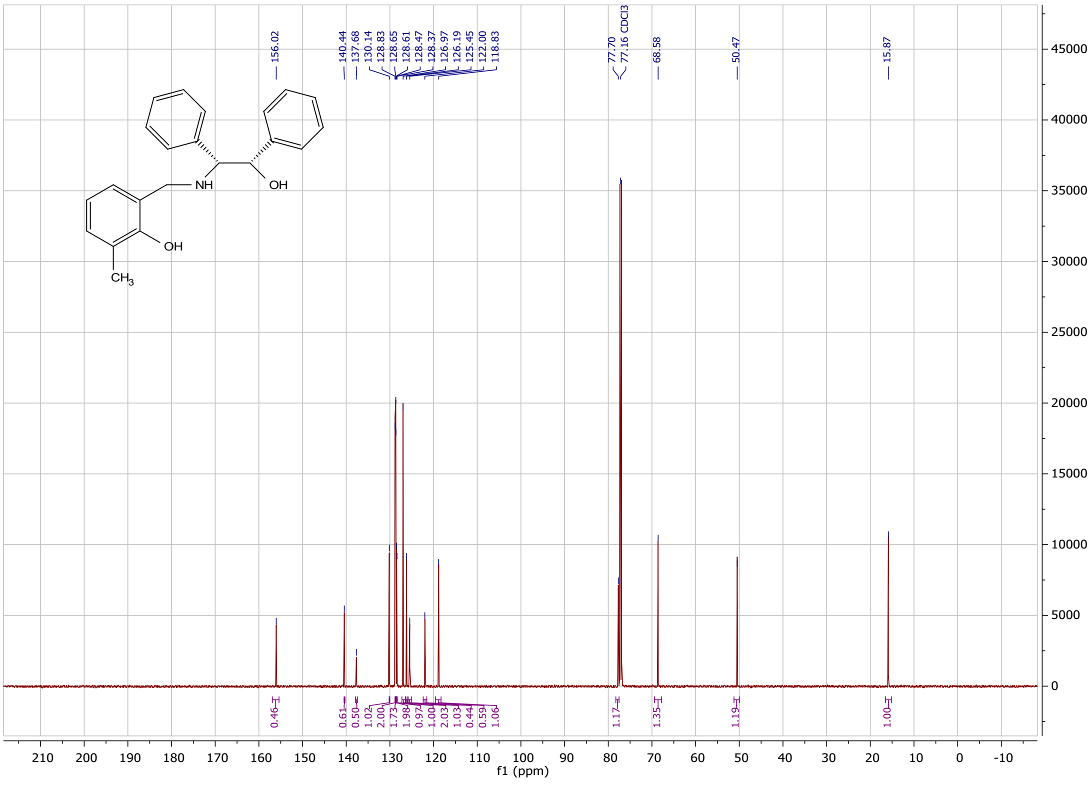

Figure S8. ^{13}C NMR spectrum of (1*S*,2*R*)-2-(N-2'-hydroxyl-3'-methylbenzyl)amino-1,2-diphenyl-1-ethanol (ligand **B**)

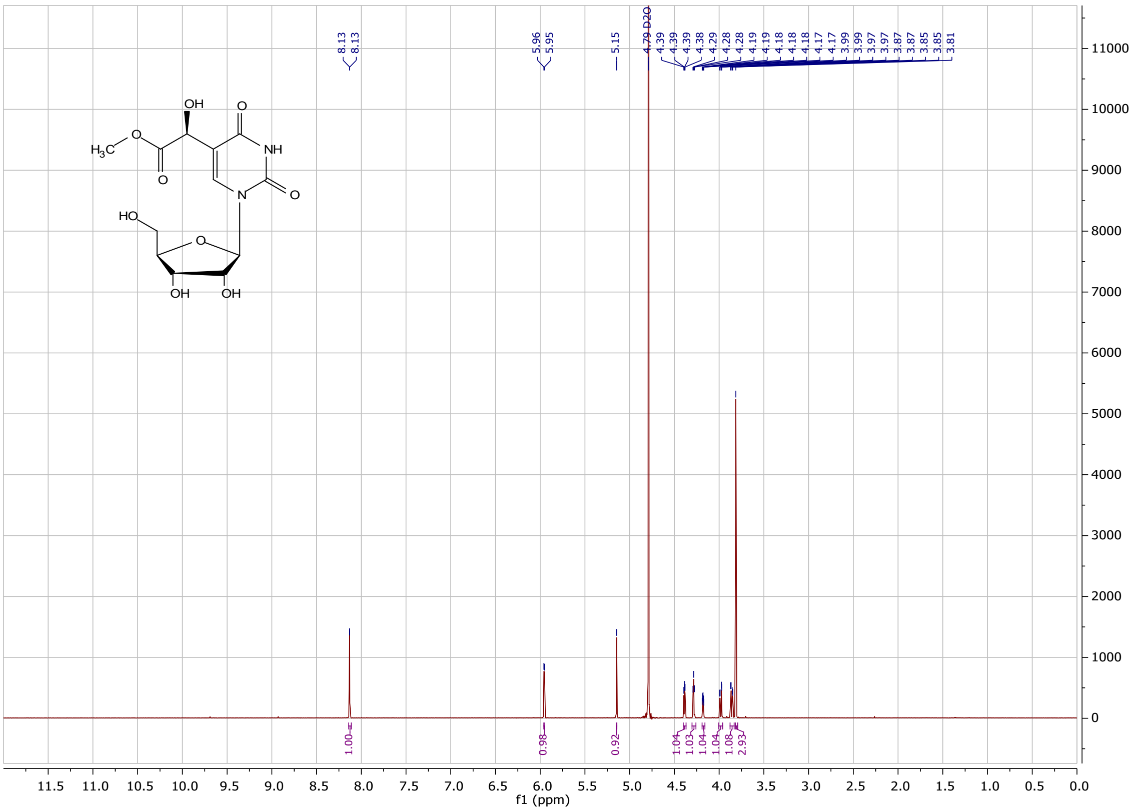

Figure S9. ^{1}H NMR spectrum of (*S*)-5-methoxycarbonylhydroxymethyluridine **1** (*S*)-mchm^{5}U

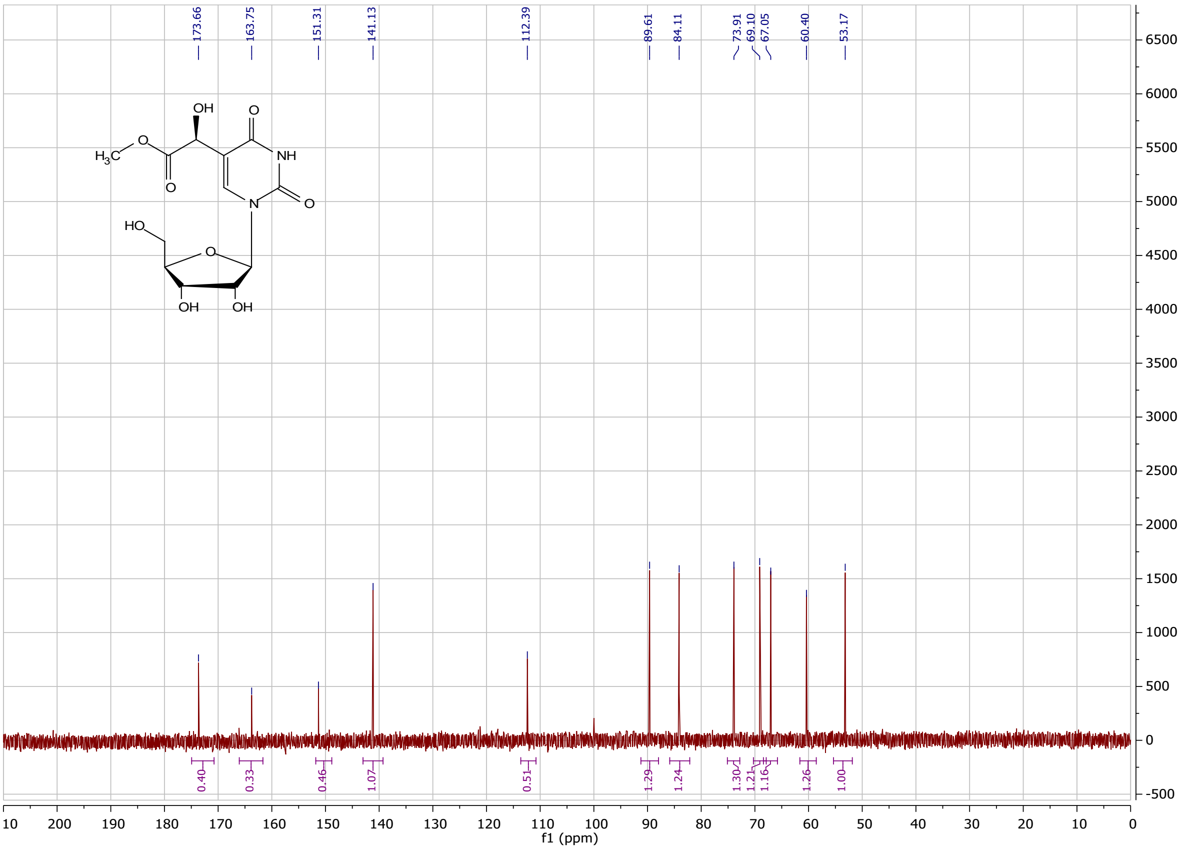

Figure S10. ^{13}C NMR spectrum of (*S*)-5-methoxycarbonylhydroxymethyluridine **1** (*S*)-mchm^{5}U.

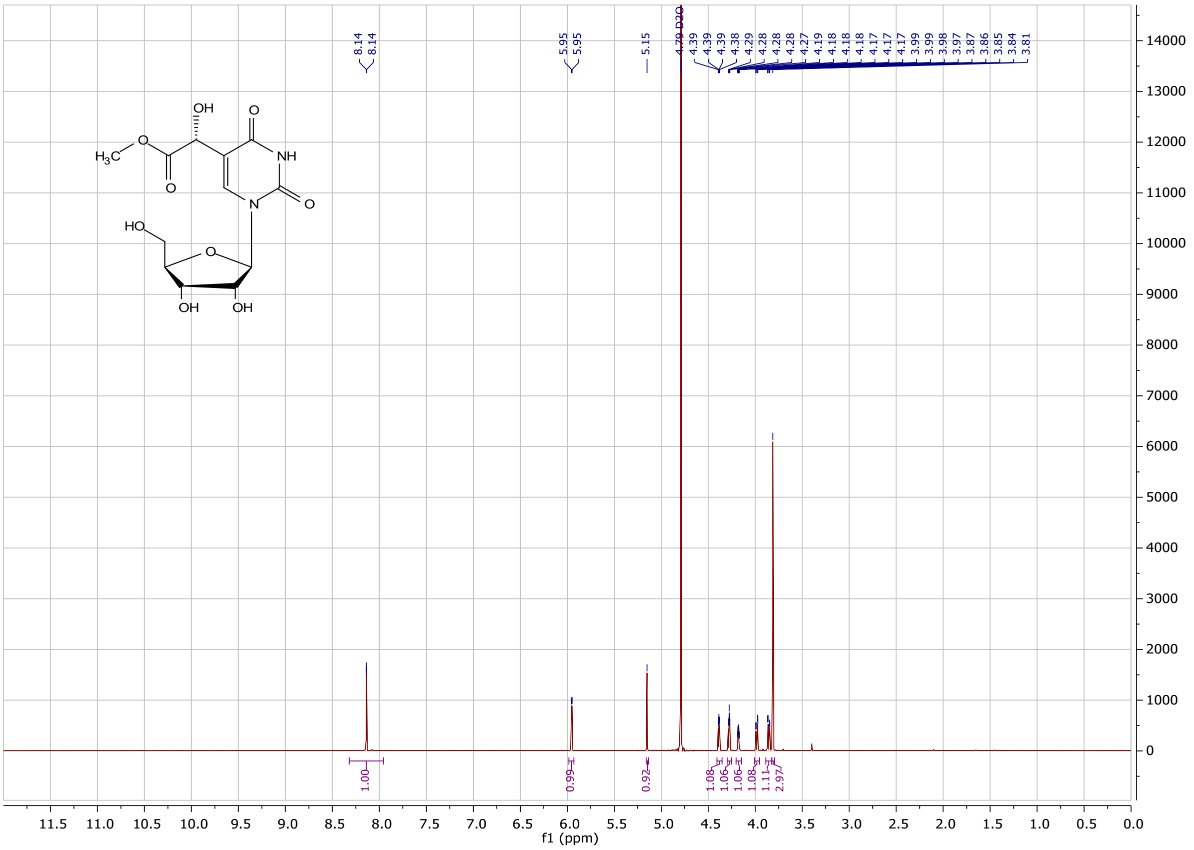

Figure S11. ^{1}H NMR spectrum of (*R*)-5-methoxycarbonylhydroxymethyluridine **2** (*R*)-mchm^{5}U

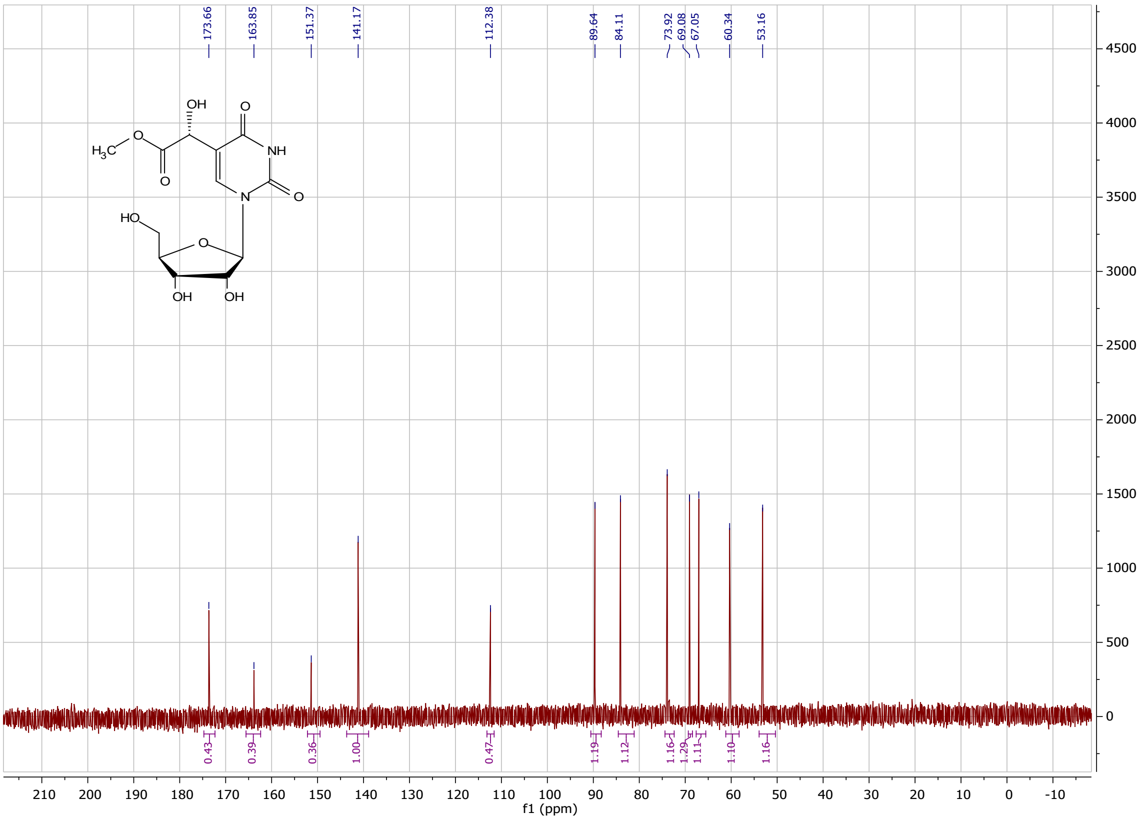

Figure S12. ^{13}C NMR spectrum of (*R*)-5-methoxycarbonylhydroxymethyluridine **2** (*R*)-mchm^{5}U

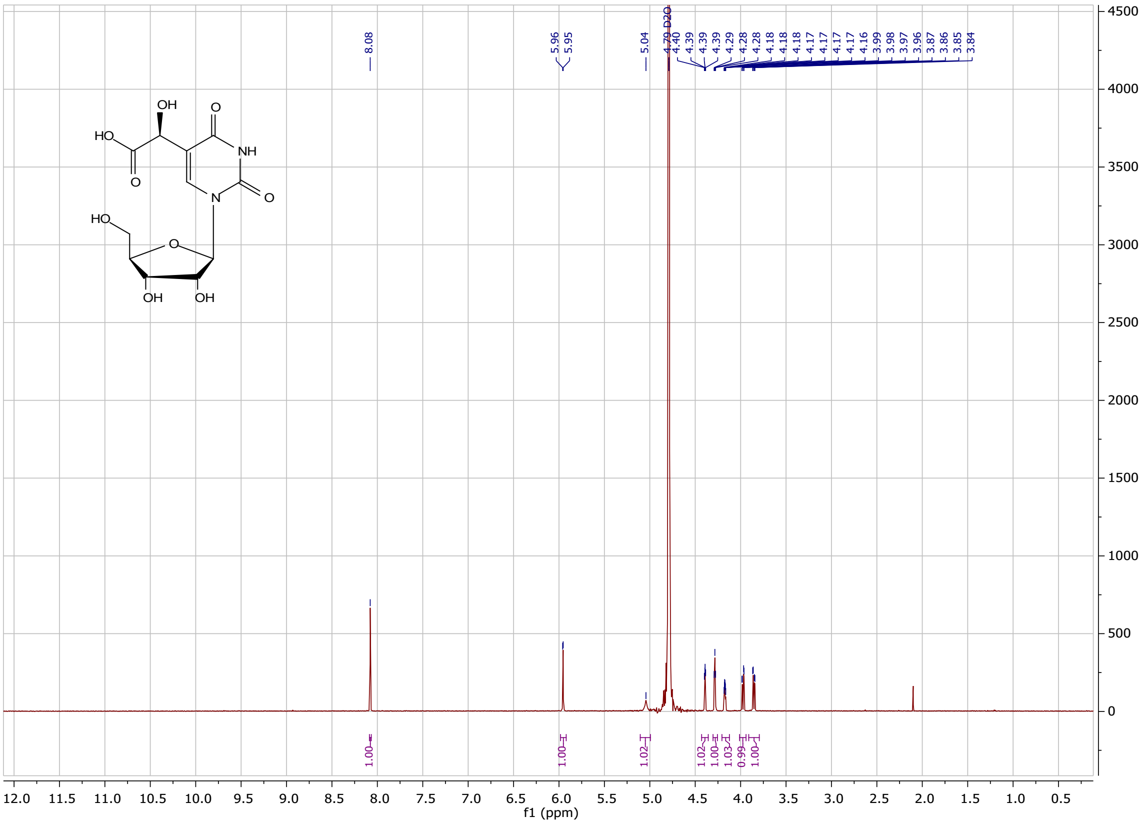

Figure S13. ^{1}H NMR spectrum of (*S*)-5-carboxyhydroxymethyluridine **5** (*S*)-chm^{5}U

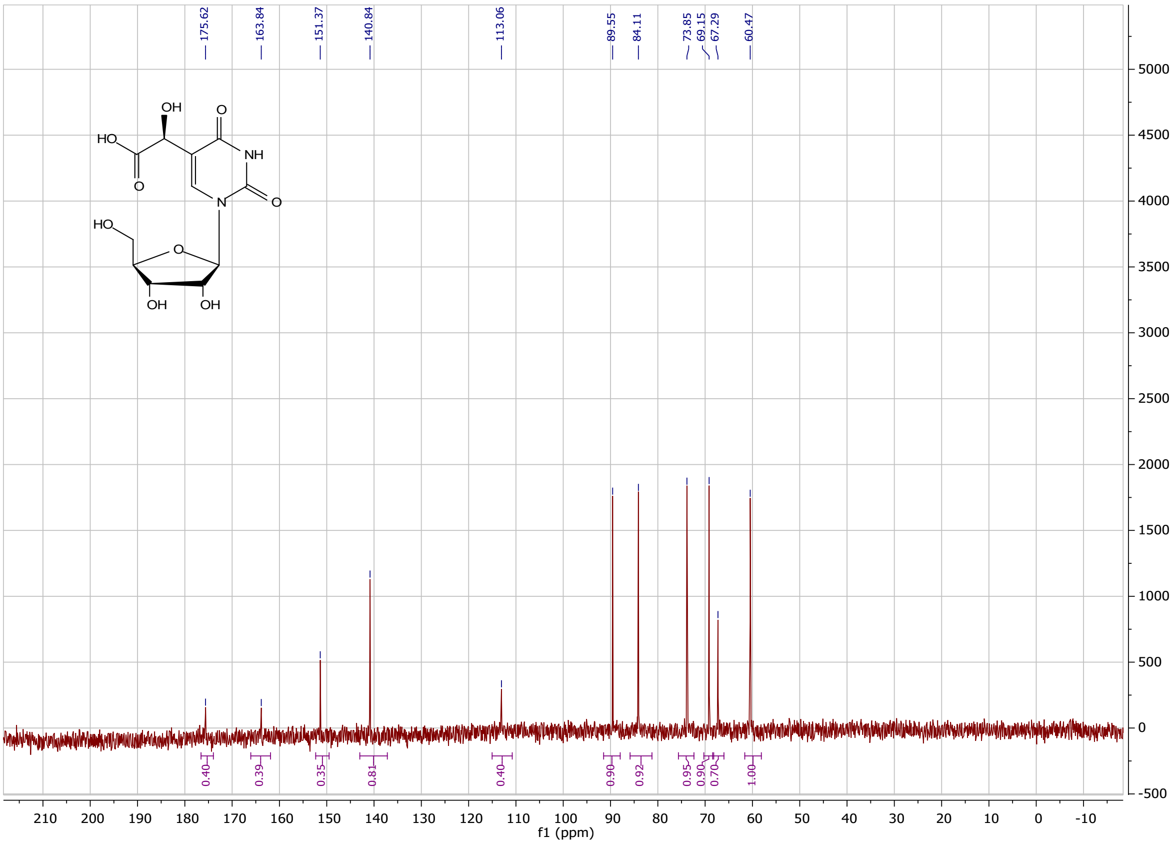

Figure S14. ^{13}C NMR spectrum of (*S*)-5-carboxyhydroxymethyluridine **5** (*S*)-chm^{5}U

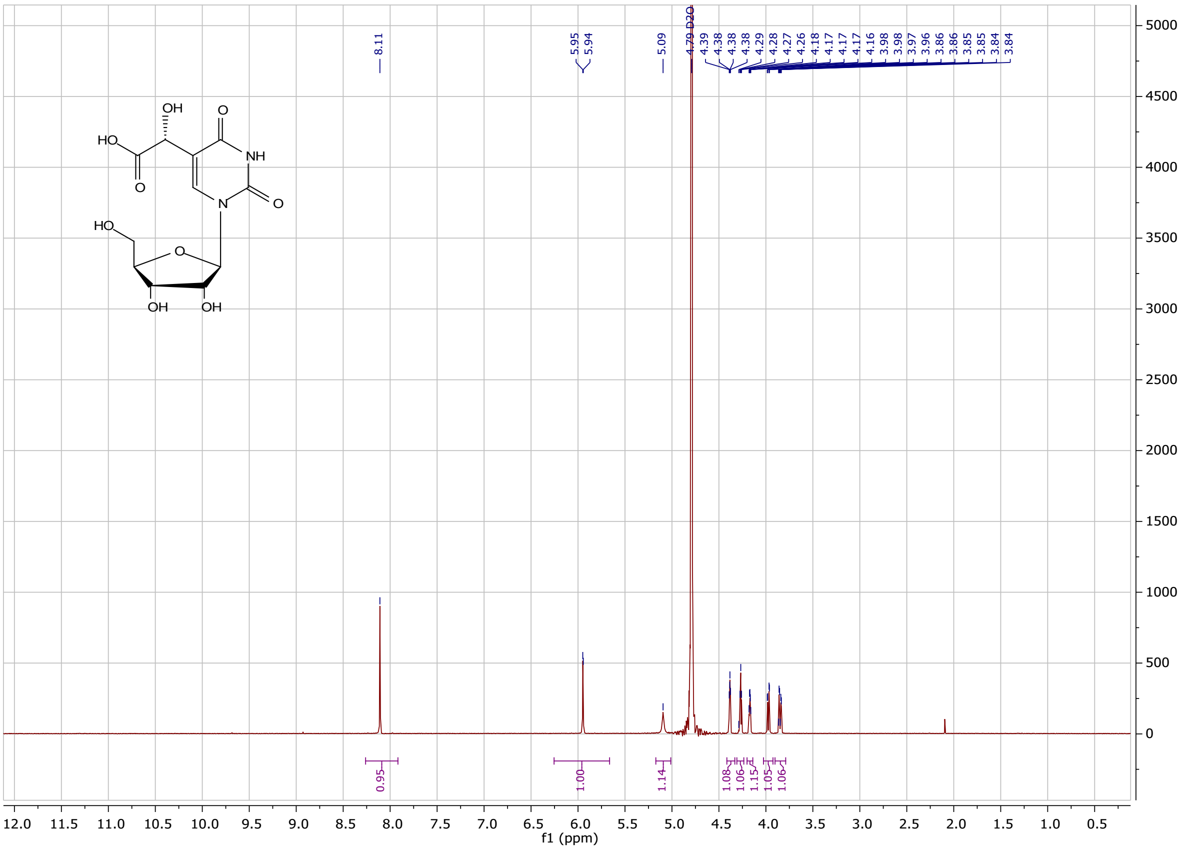

Figure S15. ^{1}H NMR spectrum of (*R*)-5-carboxyhydroxymethyluridine **6** (*R*)-chm^{5}U

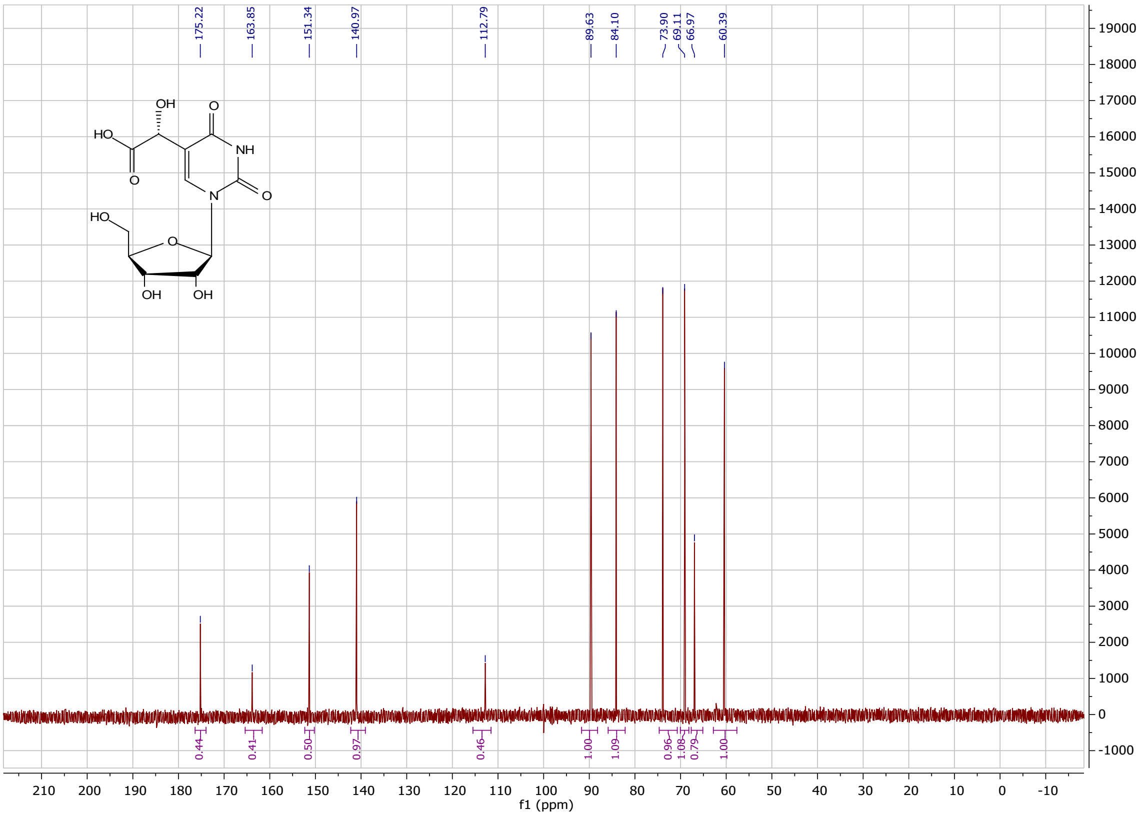

Figure S16. ^{13}C NMR spectrum of (*R*)-5-carboxyhydroxymethyluridine **6** (*R*)-chm^{5}U.

4. **HPLC chromatograms of (*S*)-5-methoxycarbonylhydroxymethyluridine 1 and (*R*)-5-methoxycarbonylhydroxymethyluridine 2**

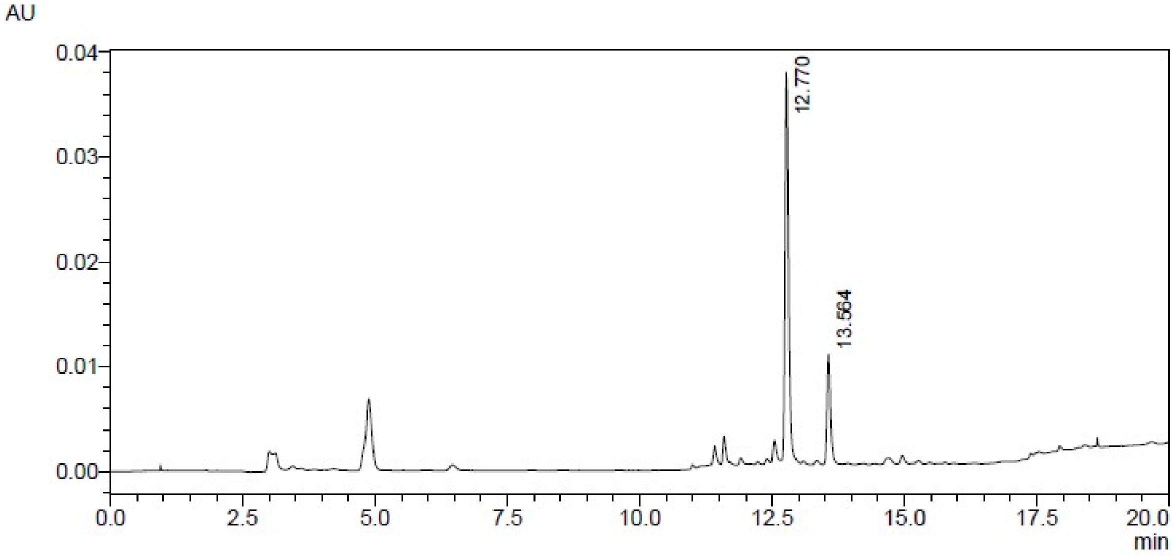

Ret. Time	Area%
12.770	78.104
13.564	21.896
	100.000

Figure S17. HPLC Chromatogram of (*S*)-5-methoxycarbonylhydroxymethyluridine **1** and (*R*)-5-methoxycarbonylhydroxymethyluridine **2** mixture directly after reaction obtained in procedure 2.5

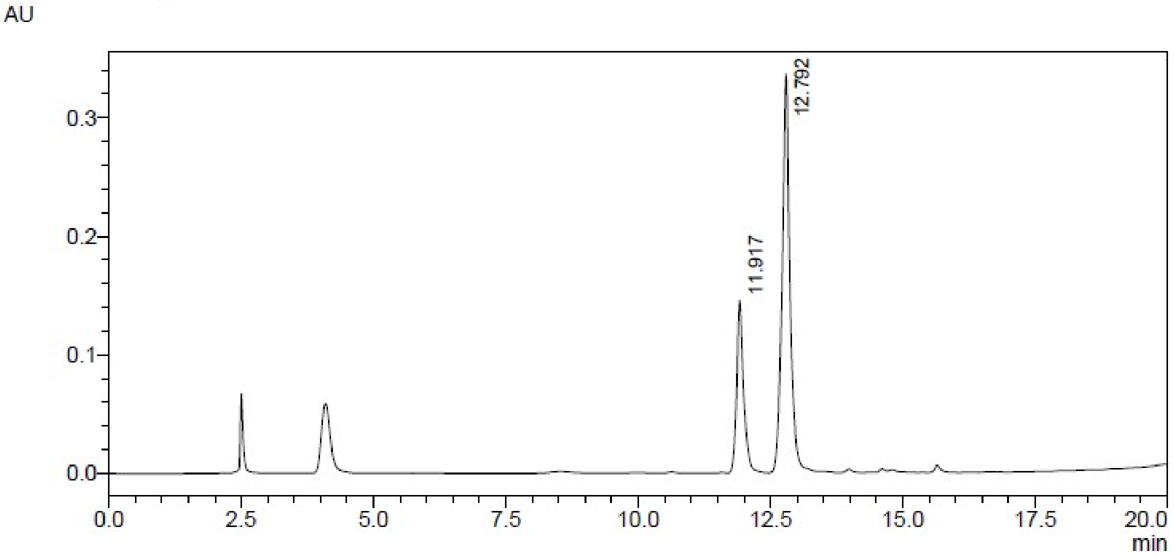

Ret. Time	Area%
11.917	25.144
12.792	74.856
	100.000

Figure S18. HPLC chromatogram of (*S*)-5-methoxycarbonylhydroxymethyluridine **1** and (*R*)-5-methoxycarbonylhydroxymethyluridine **2** mixture directly after reaction obtained in procedure 2.6

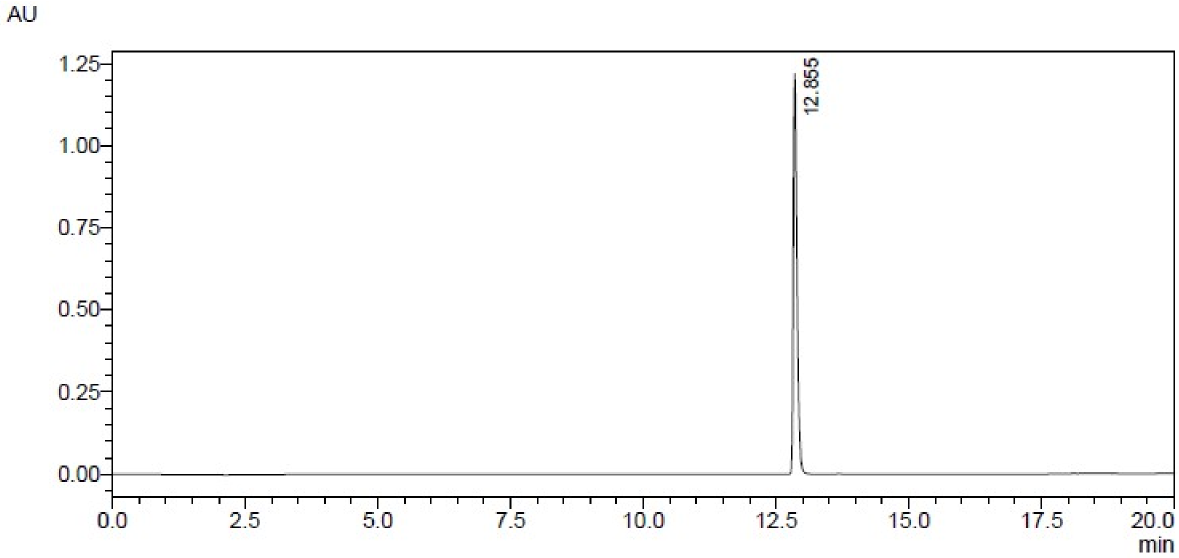

Figure S19. HPLC chromatogram of (*S*)-5-methoxycarbonylhydroxymethyluridine **1** (*S*)-mchm^{5}U after purification.

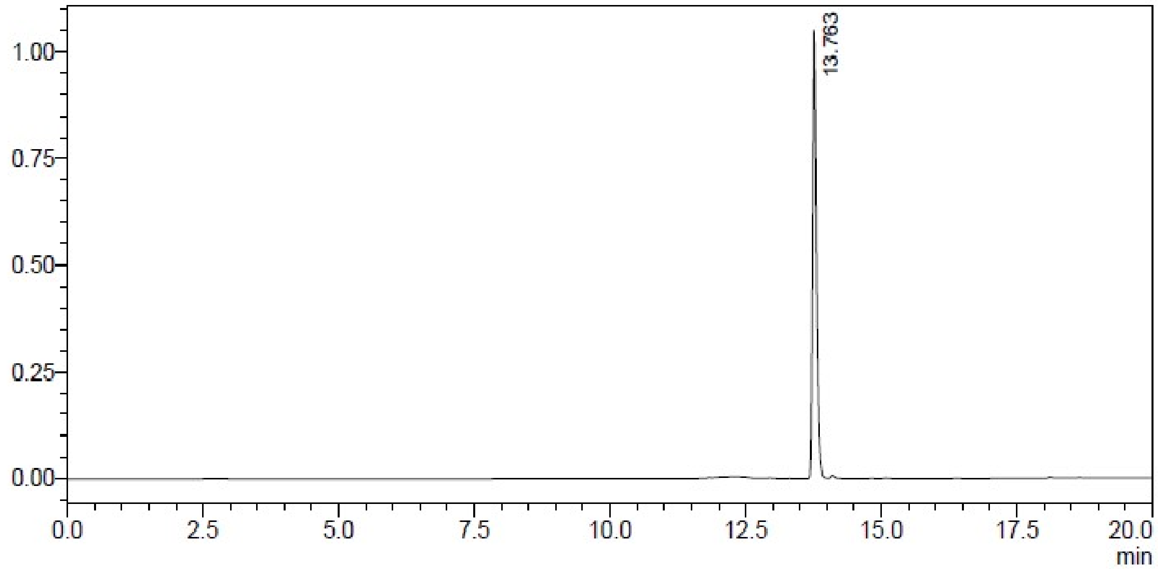

Figure S20. HPLC chromatogram of (*R*)-5-methoxycarbonylhydroxymethyluridine **2** (*R*)-mchm^{5}U after purification.

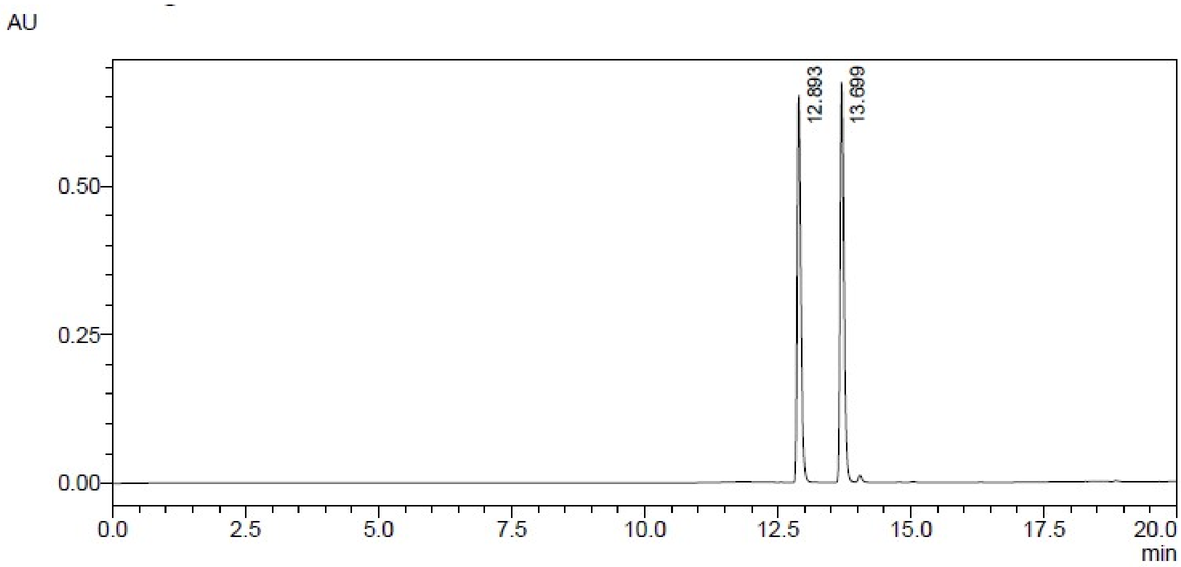

Ret. Time	Area%
12.893	49.674
13.699	50.326
	100.000

Figure S21. HPLC Chromatogram of equimolar mixture of (*S*)-5-methoxycarbonylhydroxy-methyluridine **1** and (*R*)-5-methoxycarbonylhydroxymethyluridine **2**

5.HPLC chromatograms of (*S*)-5-carboxyhydroxymethyluridine 5 and (*R*)-5-carboxyhydroxymethyluridine 6

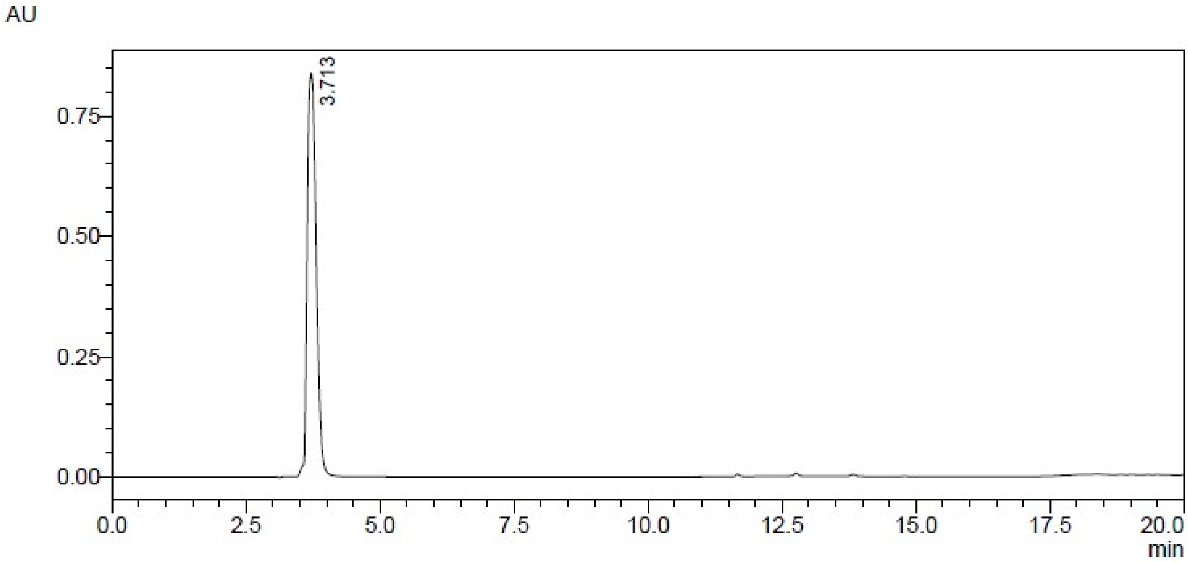

Figure S22. HPLC Chromatogram of (*S*)-5-carboxyhydroxymethyluridine **5** (*S*)-chm^{5}U after purification.

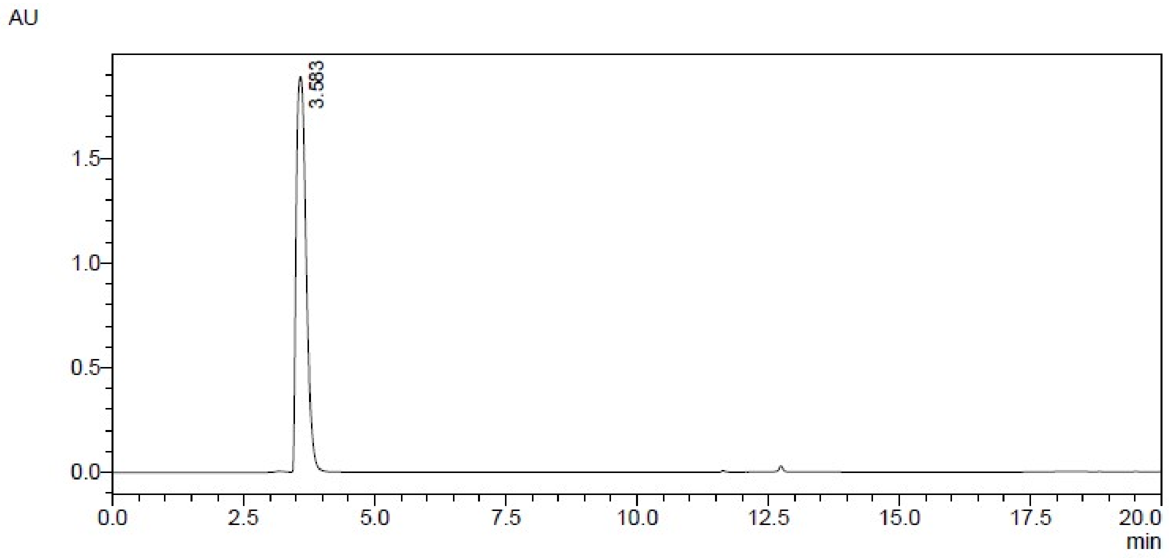

Figure S23. HPLC Chromatogram of (*R*)-5-carboxyhydroxymethyluridine **6** (*R*)-chm^{5}U after purification.

6. MS Spectra

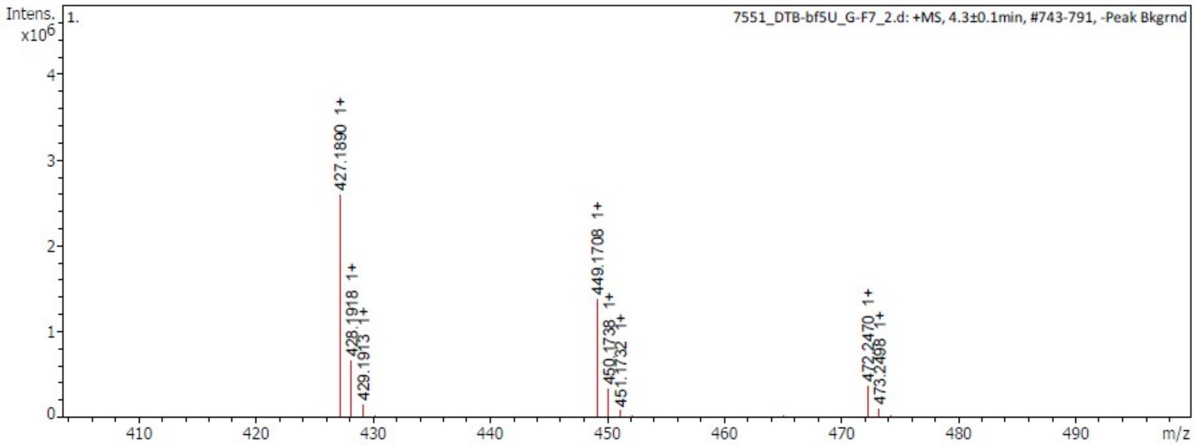

Figure S24. MS Spectrum of 5'-*O*-*tert*-butyldimethylsilyl-5-formyl-2',3'-*O*-isopropylideneuridine **8**

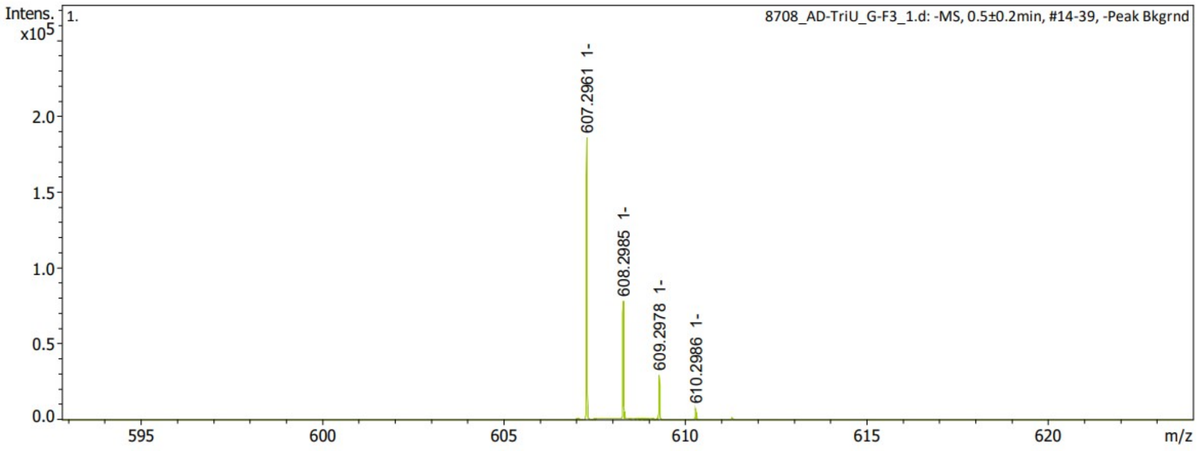

Figure S25. MS Spectrum of 2’,3’-*O*-isopropylidene-5’-*O*-*tert*-butyldimethylsilyl-5-(*tert*-butyldimethylsilyloxy)(1-*H*-imidazol-1-yl)methyluridine **9**

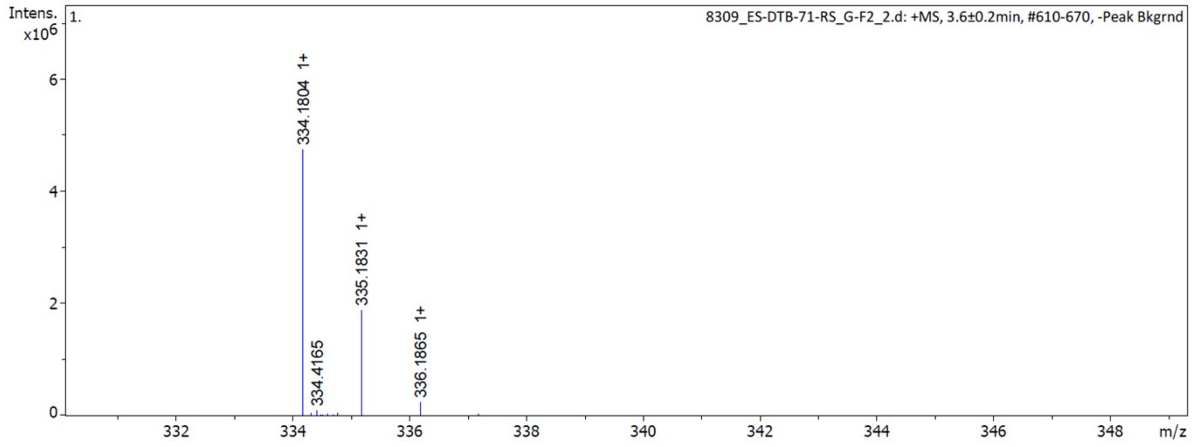

Figure S26. MS Spectrum of (1*R*,2*S*)-2-(*N*-2'-hydroxyl-3'-methylbenzyl)amino-1,2-diphenyl-1-ethanol (ligand **A**)

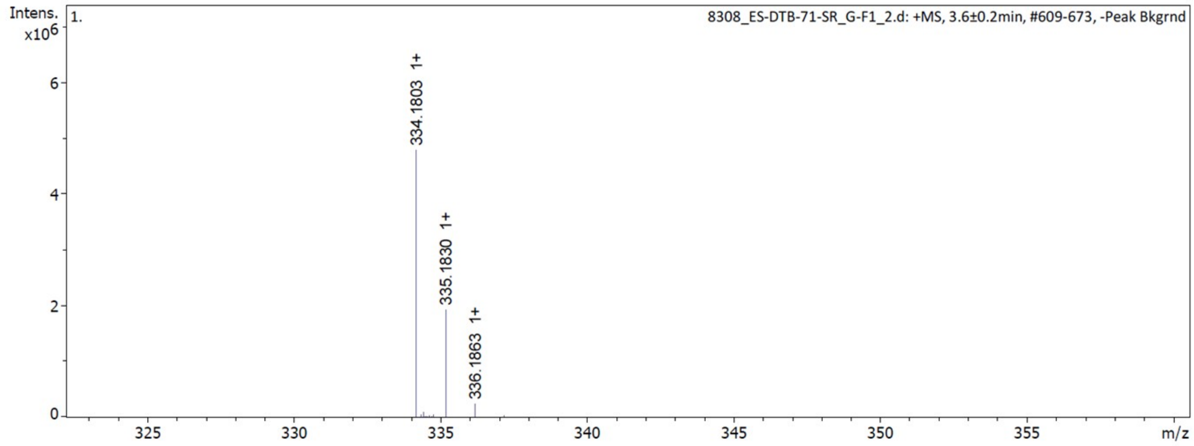

Figure S27. MS Spectrum of (1*S*,2*R*)-2-(N-2'-hydroxyl-3'-methylbenzyl)amino-1,2-diphenyl-1-ethanol (ligand **B**)

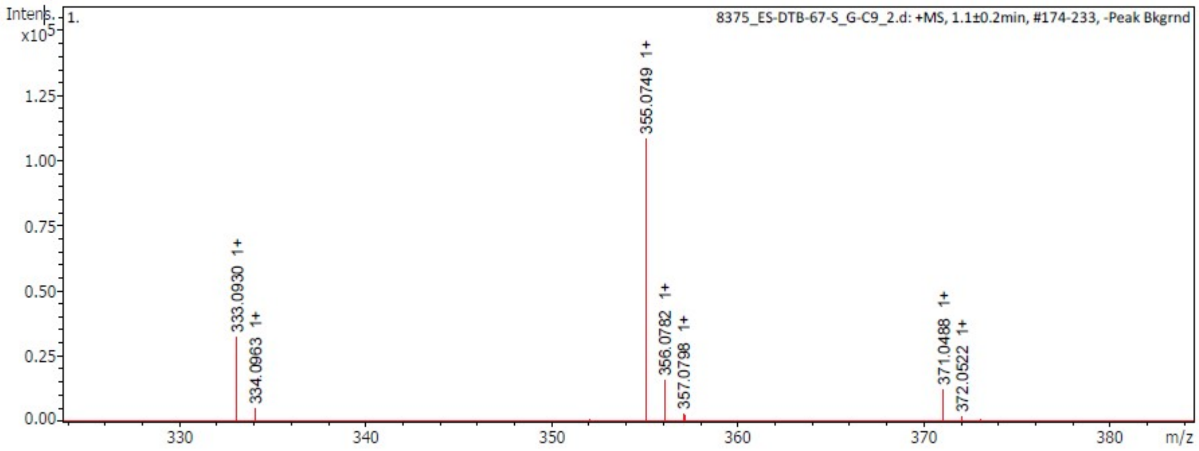

Figure S28. MS Spectrum of (*S*)-5-methoxycarbonylhydroxymethyluridine **1** (*S*)-mchm^{5}U

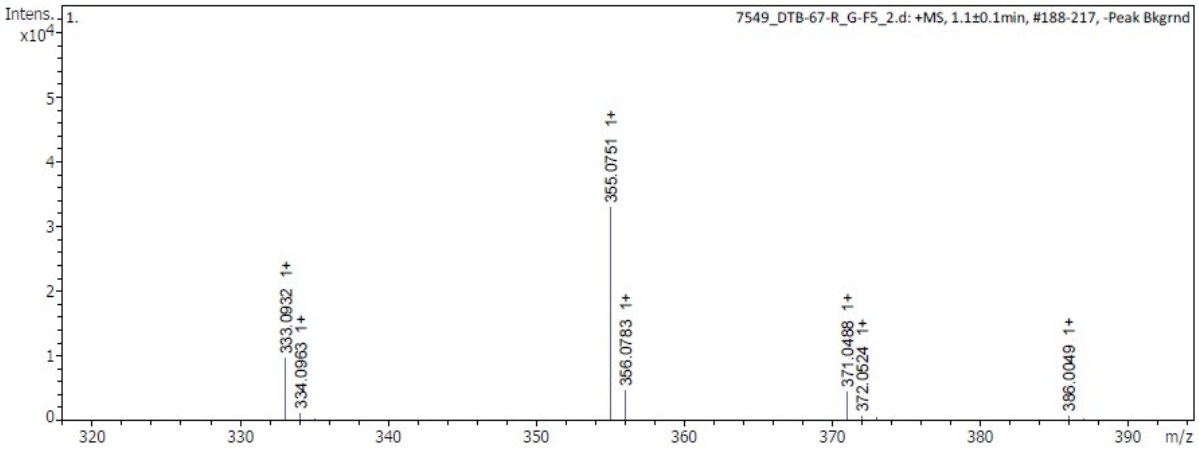

Figure S29. MS Spectrum of (*R*)-5-methoxycarbonylhydroxymethyluridine **2** (*R*)-mchm^{5}U

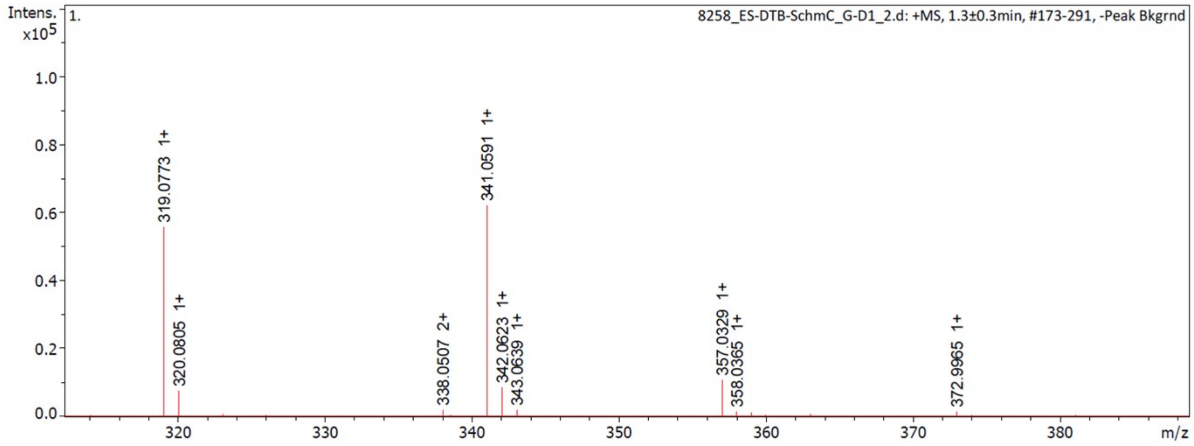

Figure S30. MS Spectrum of (*S*)-5-carboxyhydroxymethyluridine **5** (*S*)-chm^{5}U

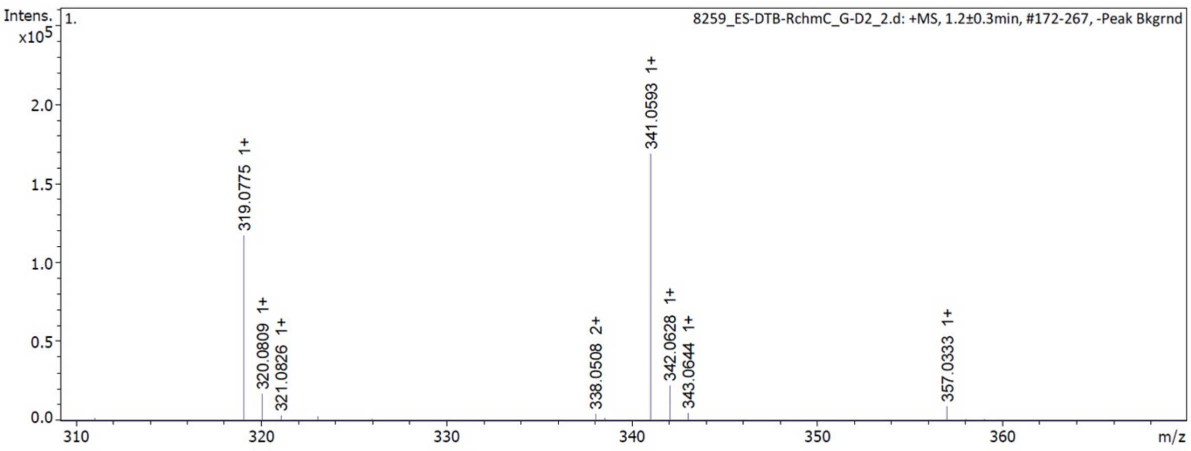

Figure S31. MS Spectrum of (*R*)-5-carboxyhydroxymethyluridine **6** (*R*)-chm^{5}U

7. CD Spectra

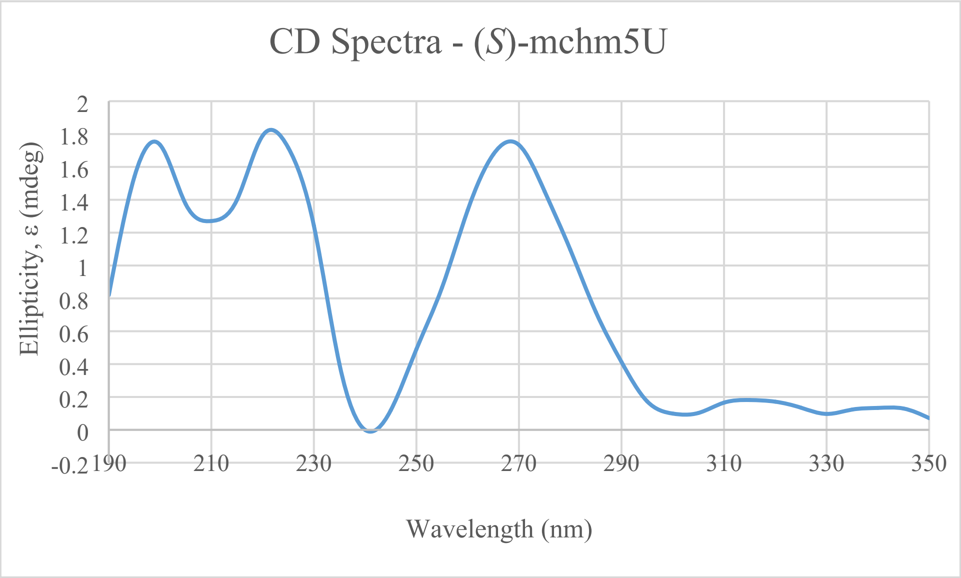

Figure S32. CD spectrum of (*S*)-5-methoxycarbonylhydroxymethyluridine **1** (*S*)-mchm^{5}U

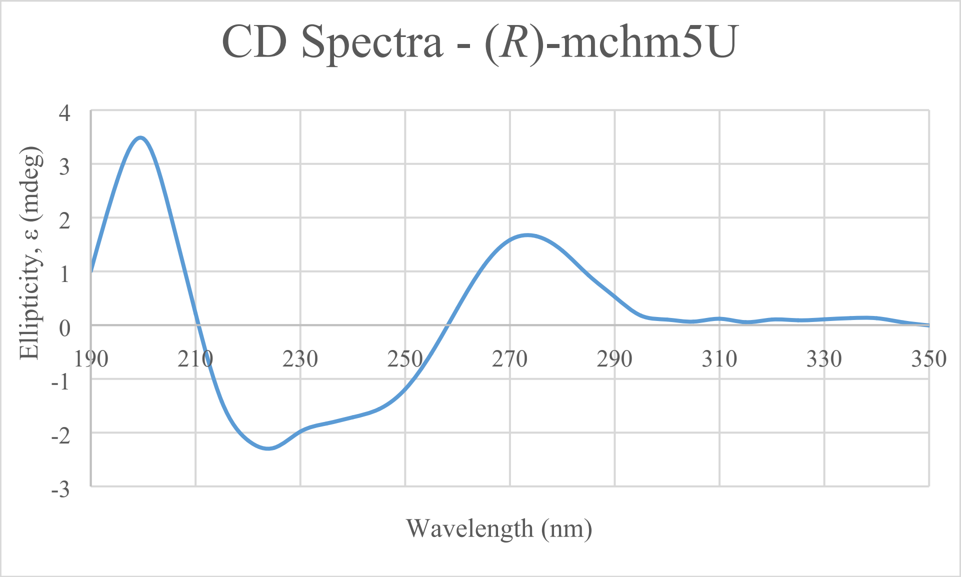

Figure S33. CD spectrum of (*R*)-5-methoxycarbonylhydroxymethyluridine **2** (*R*)-mchm^{5}U

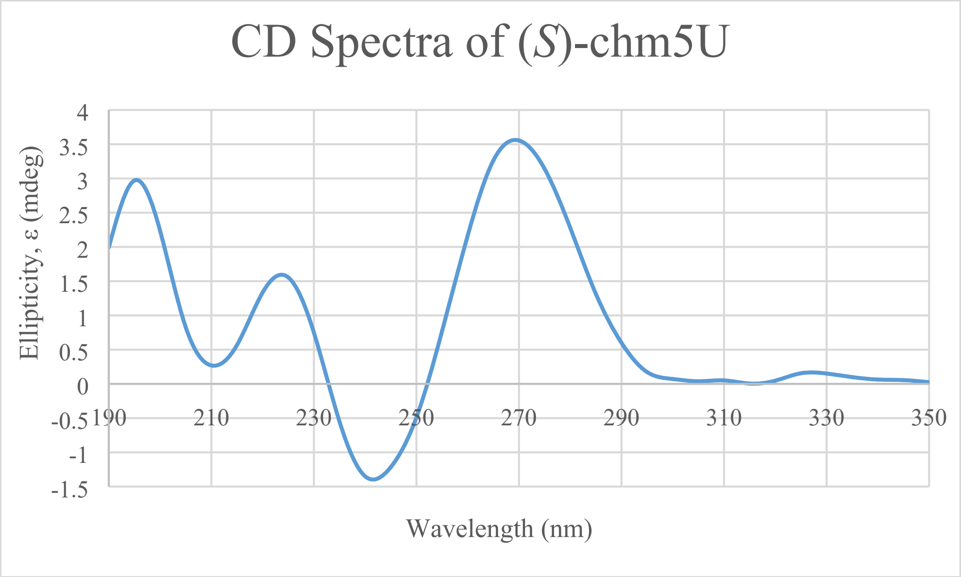

Figure S34. CD spectrum of (*S*)-5-carboxyhydroxymethyluridine **5** (*S*)-chm^{5}U

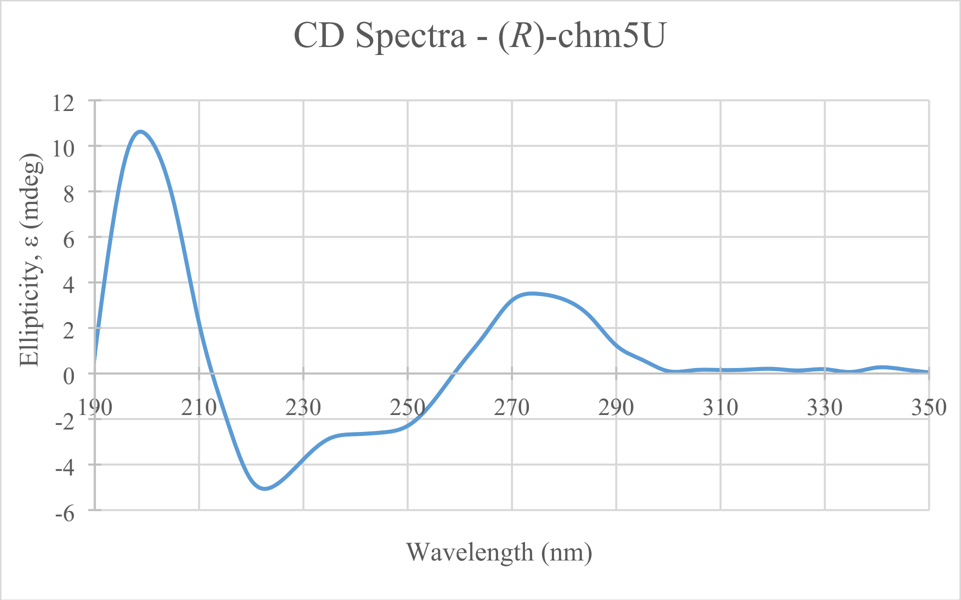

Figure S35. CD spectrum of (*R*)-5-carboxyhydroxymethyluridine **6** (*R*)-chm^{5}U

6. References

1. Y. Li, B. He, B. Qin, X. Feng and G. Zhang, *J. Org. Chem.*, 2004, **69**, 7910.
